# Functional male accessory glands and fertility in Drosophila require novel ecdysone receptor

**DOI:** 10.1371/journal.pgen.1006788

**Published:** 2017-05-11

**Authors:** Vandana Sharma, Anuj K. Pandey, Ajay Kumar, Snigdha Misra, Himanshu P. K. Gupta, Snigdha Gupta, Anshuman Singh, Norene A. Buehner, Kristipati Ravi Ram

**Affiliations:** 1 Embryotoxicology Laboratory, Environmental Toxicology Group, CSIR- Indian Institute of Toxicology Research (CSIR-IITR), Vishvigyan Bhavan, 31, Mahatma Gandhi Marg, Lucknow, Uttar Pradesh, India; 2 Academy of Scientific and Innovative Research (AcSIR), CSIR-IITR Campus, Lucknow. Uttar Pradesh, India; 3 Dept. of Molecular Biology and Genetics, Cornell University, Ithaca, NY, United States of America; Howard Hughes Medical Institute, UNITED STATES

## Abstract

In many insects, the accessory gland, a secretory tissue of the male reproductive system, is essential for male fertility. Male accessory gland is the major source of proteinaceous secretions, collectively called as seminal proteins (or accessory gland proteins), which upon transfer, manipulate the physiology and behavior of mated females. Insect hormones such as ecdysteroids and juvenoids play a key role in accessory gland development and protein synthesis but little is known about underlying molecular players and their mechanism of action. Therefore, in the present study, we examined the roles of hormone-dependent transcription factors (Nuclear Receptors), in accessory gland development, function and male fertility of a genetically tractable insect model, *Drosophila melanogaster*. First, we carried out an RNAi screen involving 19 hormone receptors, individually and specifically, in a male reproductive tissue (accessory gland) for their requirement in Drosophila male fertility. Subsequently, by using independent RNAi/ dominant negative forms, we show that Ecdysone Receptor (EcR) is essential for male fertility due to its requirement in the normal development of accessory glands in Drosophila: EcR depleted glands fail to make seminal proteins and have dying cells. Further, our data point to a novel ecdysone receptor that does not include Ultraspiracle but is probably comprised of EcR isoforms in Drosophila male accessory glands. Our data suggest that this novel ecdysone receptor might act downstream of homeodomain transcription factor paired (prd) in the male accessory gland. Overall, the study suggests novel ecdysone receptor as an important player in the hormonal regulation of seminal protein production and insect male fertility.

## Author summary

Insects are the major contributors to biodiversity and have economic, agricultural and health importance. This unparalleled abundance of insects, in part, can be attributed to their high reproductive potential. In many insects, proteins derived from the accessory gland, the secretory tissue of male reproductive system, are critical for fertility. The production of these accessory gland proteins is regulated by insect hormones but the underlying mechanisms/molecular players remain poorly understood. Elucidation of the same has potential applications in designing pest control management strategies and to understand the effect of environmental chemicals on reproduction. In view of this, we analyzed the role, if any, of various insect hormone receptors in development and function of the male accessory gland in a genetically tractable insect model, *Drosophila melanogaster*. Here, we report the involvement of Ecdysone receptor (EcR with novel composition) in Drosophila male fertility. We show that the depletion of this receptor causes cell death in male accessory glands, which fail to produce seminal fluid proteins leading to sterility/sub-fertility of Drosophila males. These findings will find potential applications in designing insect pest control strategies.

## Introduction

In insects, molecular contributions from the male play critical roles in altering the reproductive physiology and behavior of the mated female. These physiological and behavioral changes, including elevated egg production, decreased willingness to remating, storage/utilization of sperm, altered gene expression and increased feeding [reviewed in 1,2], collectively referred to as post-mating responses, are dependent on the receipt of sperm and seminal proteins [3–5]. In insects, the majority of these seminal proteins are contributed by the accessory glands, a secretory tissue of the male reproductive tract [6–11]. The accessory glands are derived from the genital disc during the late larval stage and the development, as well as the secretory activity of the male accessory gland, is tightly regulated by the insect endocrine system [12,13]. The roles of ecdysteroids and juvenoids, the major insect hormones, in maturation and for the stimulation of protein synthesis in male accessory glands of many insects are well understood [14–22]. Despite this, the molecular players and/or receptors and their mechanism of action in the hormonal regulation of male accessory gland structure and function remain poorly understood. Mutations in Methoprene-tolerant (Met, a juvenile hormone receptor; [23]), or knockdown through injection of dsRNA for two hormone receptors (E75 and DHR38; [24]) resulted in a marginal reduction in the protein synthesis in the male accessory glands. However, it is uncertain if this phenotypic outcome is due to tissue specific /systemic role(s) of these hormone receptors. Precise characterization of the underlying hormone receptors can lead to a mechanistic understanding of the hormonal regulation of insect male accessory glands useful in designing pest control management strategies and to evaluate the effect of man-made endocrine disrupting chemicals on the reproduction of economically important insects. Hence, an attempt has been made in the present study to identify the hormone receptor(s) underlying the development and/or synthetic activity of insect male accessory glands, by taking advantage of a versatile insect model system, namely *Drosophila melanogaster*.

In Drosophila, the steroid hormone ecdysone and the terpenoid juvenile hormone (JH) regulate major developmental and adult processes [25]. The male accessory glands in Drosophila develop from mesodermal cells recruited into the genital disc during late larval development [26]. Each lobe of the male accessory gland in Drosophila is composed of a monolayer of binucleate morphologically distinct cell types with 96% flat polygonally shaped “main cells” and 4% large, spherical, vacuole filled “secondary” cells [27,28]. In *D*. *melanogaster*, the programming of these cell types by homeodomain transcription factor, *paired* (*prd*), homeodomain transcription repressor, *defective proventriculus* (*dve*) and homeobox gene, *Abdominal-B* (*Abd-B*) is critical for male fertility as their concerted synthetic activities lead to production of functional accessory gland proteins (Acps; [29–33]). These Acps modulate multiple physiological and behavioral processes in females, after their transfer during mating [7,34–36]. In *D*. *melanogaster*, ecdysone and juvenile hormone titers are known to influence the synthesis of Acps [37,38]. Apart from *Met*, the *D*. *melanogaster* genome has 18 nuclear receptor genes [39] but their roles in accessory gland development, Acp synthesis and reproduction remain to be characterized. Therefore, we examined the requirement for 19 hormone receptors (18 nuclear receptors and Met) in Drosophila male fertility by employing the GAL4/UAS binary expression system with RNAi constructs, and inducible dominant-negative forms against these hormone receptors, in an accessory gland-specific manner. We demonstrate that ecdysone receptor (EcR) but not ultraspiracle (USP) is essential for functional male accessory glands, and hence critical for male fertility in Drosophila. We find elevated apoptotic marker(s) coupled with structural abnormalities and reduced seminal proteins in EcR deficient accessory glands. In addition, we show that all three isoforms of EcR are essential for male fertility in Drosophila. Together, our findings suggest a deviation from the traditional heterodimer of EcR and USP, and point to a novel receptor comprising EcR homodimers in the development/function of male accessory glands in Drosophila.

## Results and discussion

### EcR is essential for male fertility in *D*. *melanogaster*

**Fig 1:**
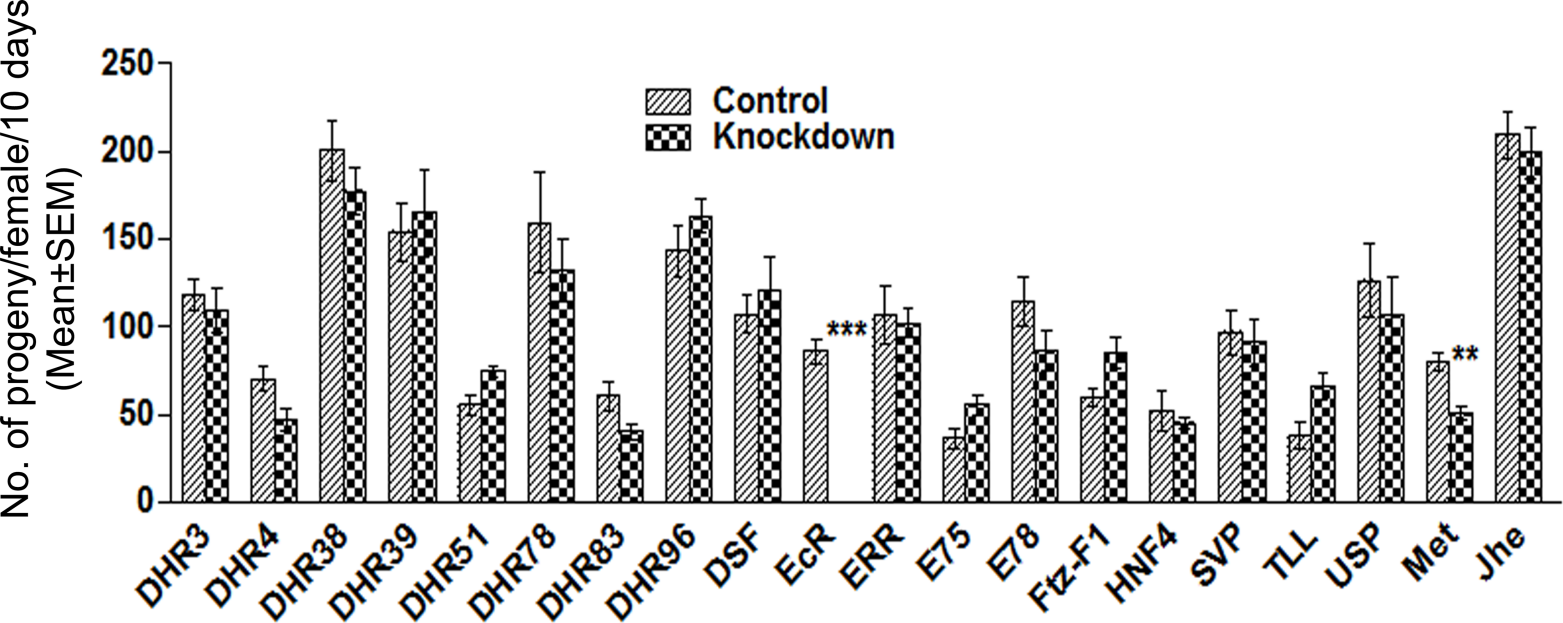
The effect of knockdown of different hormone receptors on the fertility of mated females. To identify the nuclear/hormone receptor involved in male fertility, 19 receptors were knocked down, individually, in accessory gland specific manner in the male reproductive tract and were allowed to mate with virgin females. Shown here is the number [Mean±Standard Error (SEM)] of progeny produced by females mated to knockdown or control males over a period of 10 days. Mates of *met* (Methoprene tolerant, a juvenile hormone receptor, **p<0.001) knockdown males produced significantly fewer progeny when compared to their controls and also those in strain background control (Jhe). Interestingly, females mated to EcR knockdown males failed to produce progeny (EcR; ***p<0.0001; Bonferroni corrected p value for significance is *p<*0.002). However, fertility of females mated to USP (*p =* 0.27) or the remaining 16 hormone receptor knockdown males was not significantly different from their respective controls. Number of females ranged from 15–45 depending on the hormone receptor analyzed.

**Fig 2:**
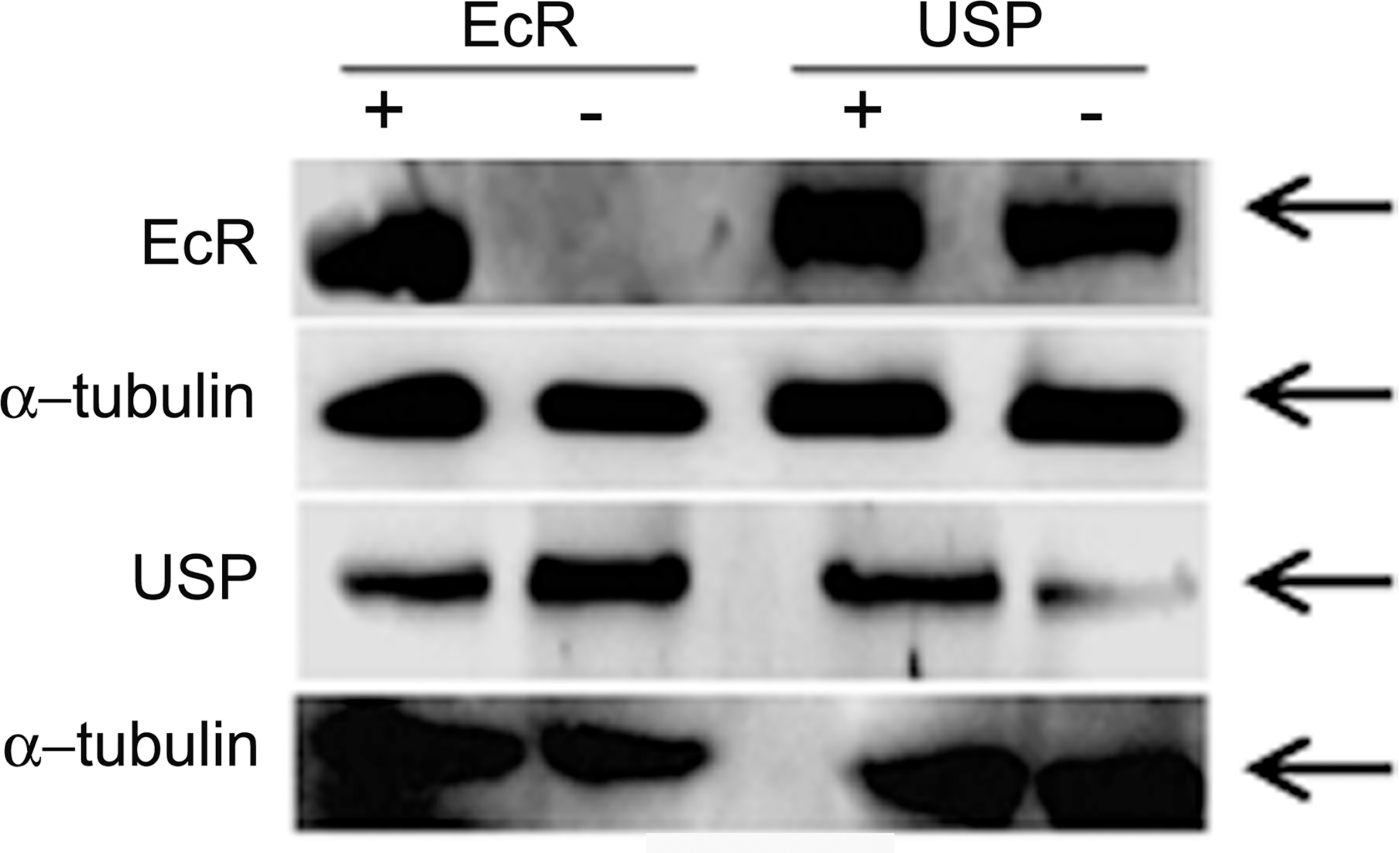
Western blots showing the levels of EcR and USP in knockdown males compared to control males. The EcR panel represents EcR levels in accessory glands from EcR control (+ lane, EcR), EcR knockdown (-lane, EcR), USP control (+ lane, USP) and USP knockdown (- lane, USP). Similarly, the USP panel represents the USP levels observed in accessory glands from above groups. Blots probed with α-tubulin antibodies (α-tubulin panels) served as controls for protein loading. Knockdowns were specific to the targeted hormone receptor. Further, the deficiency of EcR did not affect USP levels and vice-versa.

In Drosophila, ecdysone and juvenile hormones are critical for reproduction [40,41]. Further, these hormones regulate the production of seminal fluid proteins in male accessory glands [12,42]. However, knowledge of the key players underlying this hormonal control is limited. In this context, we hypothesized that nuclear receptors, which are the key regulators of hormonal action, may underlie the hormonally mediated development/function of male accessory glands. To test if this is the case, we have knocked down 18 nuclear receptors [EcR, USP, DHR38, DHR78, DHR39, DSF, DHR51, DHR3, E78, DHR96, ERR, SVP, TLL, FTZ-F1, DHR83, HNF4, DHR4, E75 (Please see S1 Fig for levels of knockdown)], a JH receptor (Met) and Juvenile hormone esterase (Jhe), individually, in an accessory gland (*prd*-GAL4; Please see S2 Fig for male accessory gland-specific expression of the GAL4 driver) specific manner, in the Drosophila male reproductive tract. To begin with, we assessed the consequences of the depletion of hormone receptors (HR) associated with ecdysone/juvenile hormone signaling on male fertility in terms of the progeny produced by females mated to these knockdown males (*prd-*GAL4/UAS-*HR-miRNA*). We counted the number of progeny produced by females mated to knockdown males and compared to those from females mated to genetically matched control males (*Sb*/UAS-*HR-miRNA*), which have normal levels of the targeted candidate. Of the candidates (18 nuclear receptors, Met, Jhe) tested, females mated to Met deficient males produced significantly fewer progeny (Fig 1, Met knockdown, *p*<0.001; Bonferroni corrected p-value for significance is *p*<0.002) when compared to controls. This is consistent with the previous report of reduced fertility of null mutants of *Met*^27^ due to reduced seminal protein levels in their accessory glands [23]. Interestingly, females mated to males with EcR-deficient accessory glands did not produce any progeny (Fig 1, EcR knockdown), and differed significantly (*p*<0.0001; Bonferroni corrected p-value for significance is *p*<0.002) from their controls (which were fertile and produced progeny at levels similar to other controls; *p* = 0.13). To rule out the influence of strain and/or off-targeting effects on the observed sterility phenotype of EcR-miRNA males, we depleted EcR in a similar manner using a different RNAi construct (EcR-TRiP) and assayed the fertility of their mates. Interestingly, females mated to EcR-TRiP knockdown males produced progeny, albeit the numbers were yet significantly lower than those of controls (S3A Fig, *p* = 0.0007). The observed discrepancy between EcR knockdown lines in the fertility phenotype may be attributed to the difference in the extent of knockdown between miRNA (97% knockdown; Fig 2) and TRiP line (33% knockdown; S3B Fig). Interestingly, knockdown of the remaining 17 nuclear receptors or Jhe did not yield detectable fertility phenotypes (Fig 1). This is interesting because many ecdysone signaling pathways in insects are known to be mediated by a heterodimer of EcR and USP [43]. Females mated to USP-deficient males, like the mates of DHR38, DHR78, DHR39, DSF, DHR51, DHR3, E78, DHR96, ERR, SVP, TLL, FTZ-F1, DHR83, HNF4, DHR4, or E75 knockdown males, did not significantly differ from their control in the overall number of progeny produced over a period of 10 days (Fig 1, USP, *p* = 0.27). One possible explanation for this lack of phenotype could be that the extent of knockdown may not be sufficient as the level of protein loss in USP is not as severe as that of EcR through miRNA based silencing. At this juncture, it is important to note that the observed knockdown levels (> 50% knockdown, S4 Fig) achieved with USP-miRNA were still better than EcR-TRiP based silencing (which was only 33% but yet resulted in a phenotype). In addition, similar depletion of USP with different additional RNAi constructs (USP-TRiP or USP-RNAi) also did not have a significant effect on male fertility: females mated to these knockdown males had fertility comparable to controls (S5 Fig). Nonetheless, if EcR is acting with USP, then one would predict that simultaneous depletion of USP shall further reduce the fertility of the weaker EcR (EcR-TRiP) knockdown. However, the number of progeny from the females mated to EcR-TRiP/USP-miRNA double knockdown males was comparable to that of females mated to EcR-TRiP knockdown males (S6 Fig; p = 0.2589). In addition, we observed that knockdown of EcR did not affect the USP levels in male accessory gland and vice-versa: EcR knockdown male accessory glands had USP protein at levels comparable to controls (Fig 2) while USP-deficient accessory glands had reduced level of USP protein but contained normal levels of EcR (Fig 2). The above findings raised the possibility that EcR may function without USP in ecdysone signaling pathways operating in/on male accessory glands. To test if this is the case, we focused on EcR and USP for further experiments.

**Fig 3:**
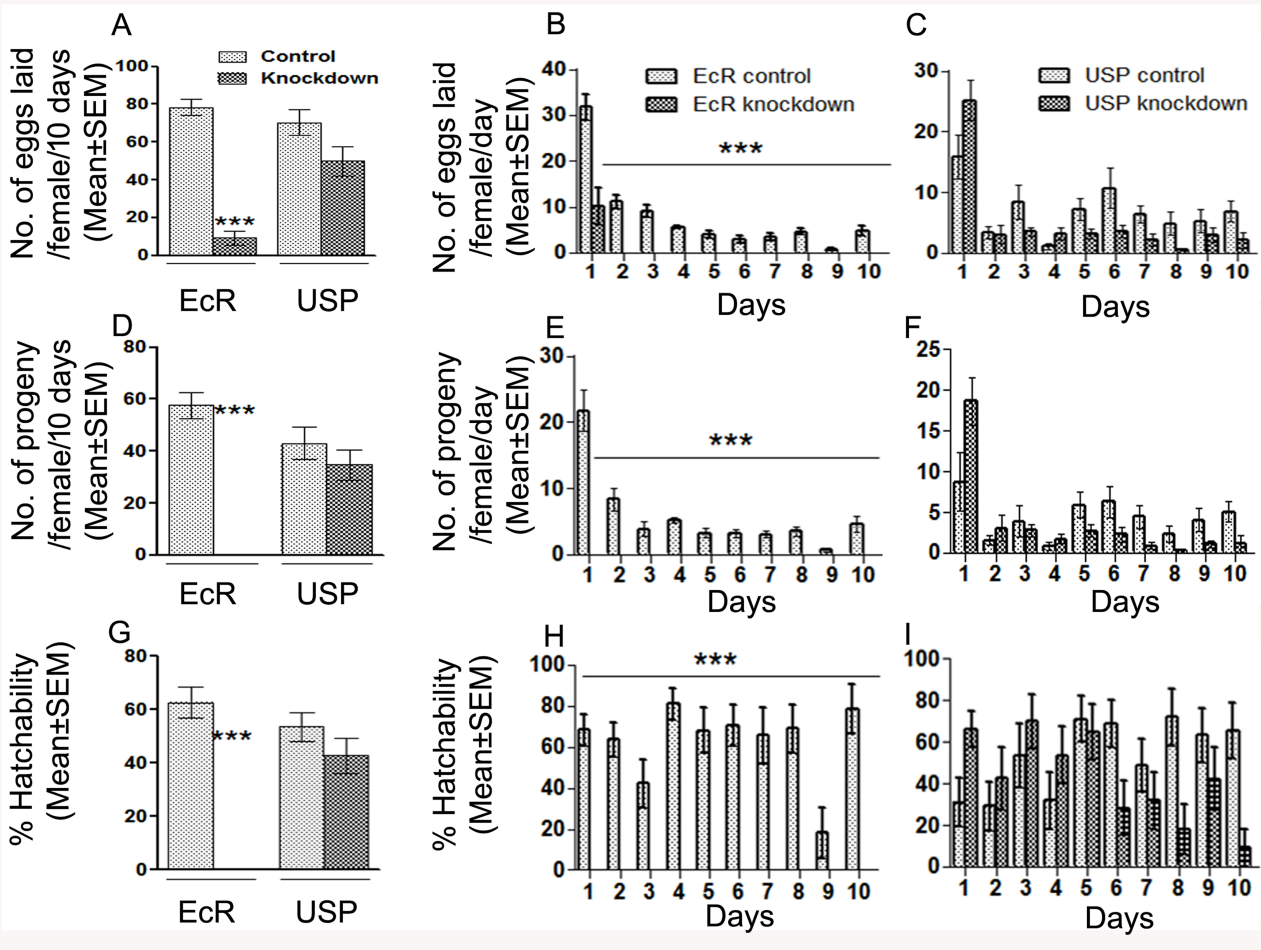
Reproductive performance of females mated to EcR or USP knockdown males over period of 10 days. Panel A represents the overall fecundity (total no. of eggs laid/10 days) of mated females while Panels B and C represent day wise fecundity (no. of egg laid/day from day1-10) of mated females. The overall fecundity of EcR knockdown mates was significantly lower compared to controls (***p<0.0001). However, overall fecundity of USP knockdown mates was comparable to their controls. (*p* = 0.08). Further, EcR knockdown mates laid eggs for 24hr ASM, albeit at significantly fewer numbers (***p<0.0001) when compared to control and did not lay eggs from days 2–10. USP knockdown mates did not deviate from controls on day wise egg laying. Interestingly, there were no progeny from the eggs laid by EcR knockdown mates over period of 10 days (overall fertility, ***p<0.0001, Panel D; day-wise fertility, ***p<0.0001 Panel E). USP knockdown or control mates had comparable overall (Panel D; *p =* 0.3, USP) as well as day wise fertility (Panel F; *p =* 0.06 lowest) EcR control and knockdown mates differ significantly on total % hatchability (Panel G; EcR, ***p<0.0001) as well as % hatchability on day 1 (Panel H; ***p<0.0001). USP control and knockdown mates had significant differences in % hatchability on days 8 and 10 (Panel I; *p* = 0.01) but that did not have a significant bearing on the overall % hatchability (Panel H; *p* = 0.3). Values given here are Mean±SEM involving at least 15–30 females depending on the hormone receptor.

In Drosophila, a reduction in fertility could be due to several reasons, including fewer eggs being laid by mated females and/or reduced hatchability/increased mortality of these laid eggs during development [36]. Therefore, to determine the cause of reduction in the fertility of mates of EcR knockdown males, we counted the number of eggs laid (fecundity), and the number of progeny (fertility) produced over a period of 10 days, by females mated to EcR or USP knockdown males. We also examined the egg hatchability and/or mortality during development by determining percent hatchability as a proportion of eggs reaching adult stage as in Ravi Ram and Wolfner [36]. Mates of EcR or USP control males or USP knockdown males laid eggs at similar levels (Fig 3A–3C, *p* = 0.1). However, females mated to EcR knockdown males laid significantly fewer eggs compared to their control mates (Fig 3A, EcR knockdown, *p*<0.0001). Mates of EcR knockdown males laid eggs within 24h after the start of mating (ASM), albeit significantly lower in number when compared to those of control mates (Fig 3B, day 1, *p*<0.0001). However beyond 24h ASM, EcR knockdown mates did not lay eggs, while control mates continued to do so (Fig 3B, Days 2–10, *p*<0.0001). Subsequently, we counted the number of flies that emerged from these vials to determine the fertility. Females mated to EcR, USP control males or USP knockdown males produced progeny at comparable levels (Fig 3D–3F, *p* = 0.1), whereas mates of EcR knockdown males had no progeny over a period of 10 days (Fig 3D & 3E, *p*<0.0001). We calculated the percentage hatchability as a proportion of eggs reaching adulthood. None of the eggs laid by females mated to EcR knockdown males (EcR-miRNA) hatched, as evidenced by the lack of larvae as well as progeny. The overall (Fig 3G, EcR knockdown, *p*<0.0001) as well as day-wise (Fig 3H, *p*<0.0001) percent hatchability levels obtained in EcR knockdown scenario were significantly lower than those in control. A similar trend, with the exception of sterility phenotype, was observed in mates of EcR-TRiP knockdown males (S7 Fig). However, USP control and knockdown mates had significant differences in percent hatchability on days 8 and 10 (Fig 3I; *p* = 0.01) but such differences did not have a significant bearing on the overall percent hatchability (Fig 3H; *p* = 0.3). Therefore, the reduced fertility of EcR knockdown mates is a consequence of reduced egg production in addition to egg hatchability. These findings suggest that normal levels of EcR in the male accessory glands are critical for male fertility.

### EcR is critical for the normal accessory glands in Drosophila male reproductive tract

**Fig 4:**
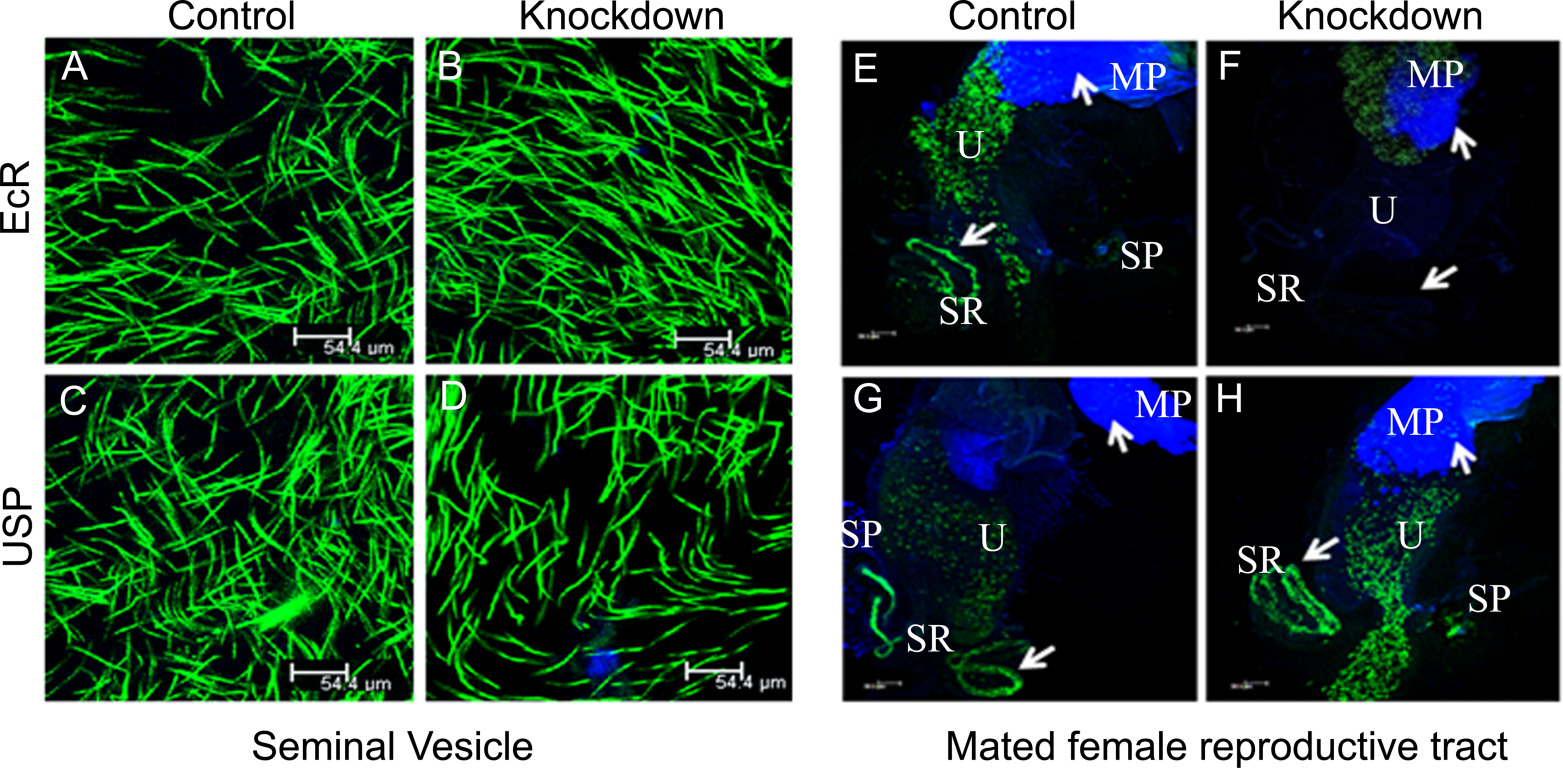
Analysis of sperm production in EcR knockdown males and their fate in mated females. To evaluate the effect of EcR knockdown (EcR-miRNA) on sperm, we first generated control and knockdown males that express GFP labeled sperm (ProtamineB-EGFP). Subsequently, observation of seminal vesicles from these males under a confocal microscope revealed comparable levels of GFP tagged sperm (green) in both controls (Panels A&C) as well as knockdown (EcR, Panel B or USP, Panel D) males, suggesting that sperm production is normal in these males. To test if these males are able to transfer sperm to females during mating, males knockdown for EcR or USP in their accessory glands were allowed to mate with Oregon-R virgin females. At 2h ASM, reproductive tracts from mated females were isolated and observed under confocal microscope for the presence of GFP-labeled sperm. Reproductive tracts from females mated to (E) EcR control, (G) USP control or (H) knockdown males contained mating plug (MP, blue) in the uteri (U) and GFP-labeled sperm (green) in the uteri as well as sperm storage organs, namely seminal receptacle (SR) and spermathecae (SP). However, reproductive tracts of females mated to EcR knockdown males (F) contained mating plugs but had sperm only in the uterus but not in SR and SP. These observations suggest that knockdown of EcR or USP has no detectable effect on sperm transfer but the sperm transferred by EcR knockdown males fail to move towards sperm storage organs and are not stored at levels comparable to those in controls.

Transfer of sperm and seminal fluids from the male to the female during mating and post-copulatory storage as well as subsequent utilization of sperm in specialized organs (seminal receptacle and spermathecae) of the mated female reproductive tract are critical determinants of fertility [1,44]. Therefore, we analyzed sperm production in knockdown males and transfer of sperm to their mates. We observed mature sperm in the seminal vesicles of EcR control (Fig 4A) and knockdown (Fig 4B) males or USP control (Fig 4C) and knockdown (Fig 4D) males, indicating the normal sperm production. This is not surprising since the *prd* driver in the study does not target testes and/or processes related to sperm production. To find out the transfer of sperm and their storage in EcR or USP knockdown mates, reproductive tracts from these females were dissected at 2h ASM and we observed that mates of EcR control (Fig 4E), USP control (Fig 4G) or knockdown (Fig 4H) males had mating plug in the uterus and GFP-tagged sperm in their uteri as well as sperm storage organs, namely seminal receptacle and spermathecae. However, females mated to EcR knockdown males contained GFP-labeled sperm only around the mating plug in their uteri (Fig 4F). Consistent with this, GFP-labeled sperm were visible in the seminal receptacles from EcR control, USP control or USP knockdown mates while seminal receptacles from EcR knockdown mates had no detectable GFP-labeled sperm (S8A–S8D Fig). Accordingly, the number of sperm stored in the sperm storage organs was significantly different between EcR control and knockdown mates (S8E Fig, EcR, *p*<0.0001) while USP knockdown mates stored sperm at levels comparable to USP controls (S8E Fig, USP, SR *p* = 0.1 and SP *p* = 0.25). These findings suggest that the knockdown of EcR and USP has no detectable effect on sperm transfer to the female. However, sperm transferred from EcR knockdown males fail to move towards storage organs and accordingly, were not able to enter storage organs. This is consistent with the reports that the movement of sperm in the female reproductive tract, their entry into- and release from- sperm storage organs require normal levels of accessory gland proteins (Acps) [1,5,7,34,35,45–47]. Therefore, the observed failure of sperm from EcR knockdown males to enter the sperm storage organs after their transfer to females points to defects in Acp content. Nuclear receptors are known to play key roles in the regulation of organogenesis and development in Drosophila [48–50]. This prompted us to analyze the accessory glands at the structural level and the production of Acps at the transcript as well as protein levels in EcR-deficient males.

**Fig 5:**
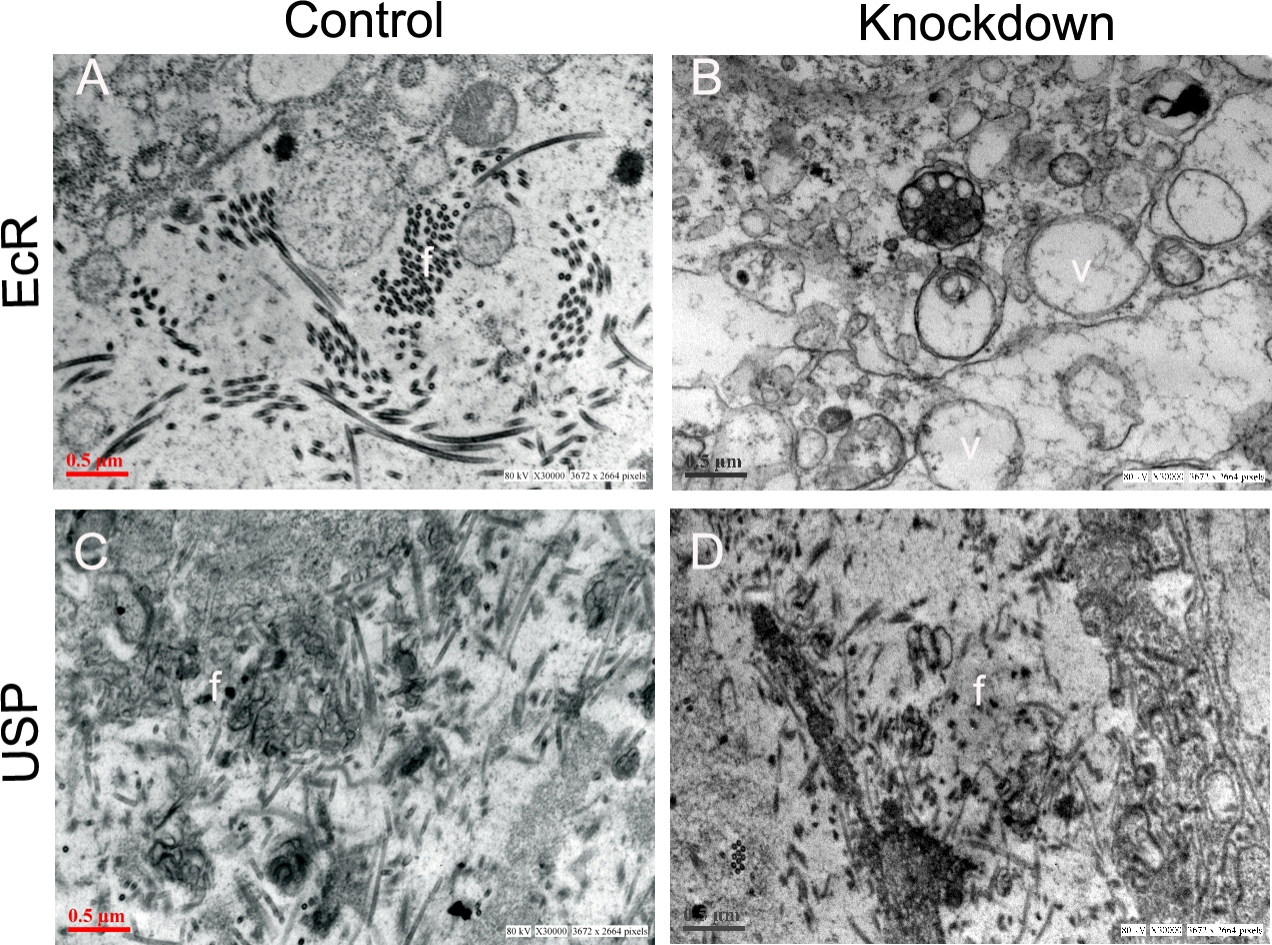
The effect of knockdown of EcR or USP on the structure of male accessory glands. To assess the effect of depletion of EcR or USP, accessory glands were analyzed either at the ultrastructural level (panels at the top). Depicted at the top are the electron micrographs of male accessory glands from (A) EcR control (B) EcR knockdown (C) USP control and (D) knockdown males. Accessory glands from EcR control, USP control and USP knockdown males show normal protein filamentous structures (labeled as f) throughout in their lumen. However, glands of EcR knockdown have extreme vacuolization (v) and lack filamentous structures in the lumen. A minimum of five tissues from each group was used for ultrastructural analysis.

**Fig 6:**
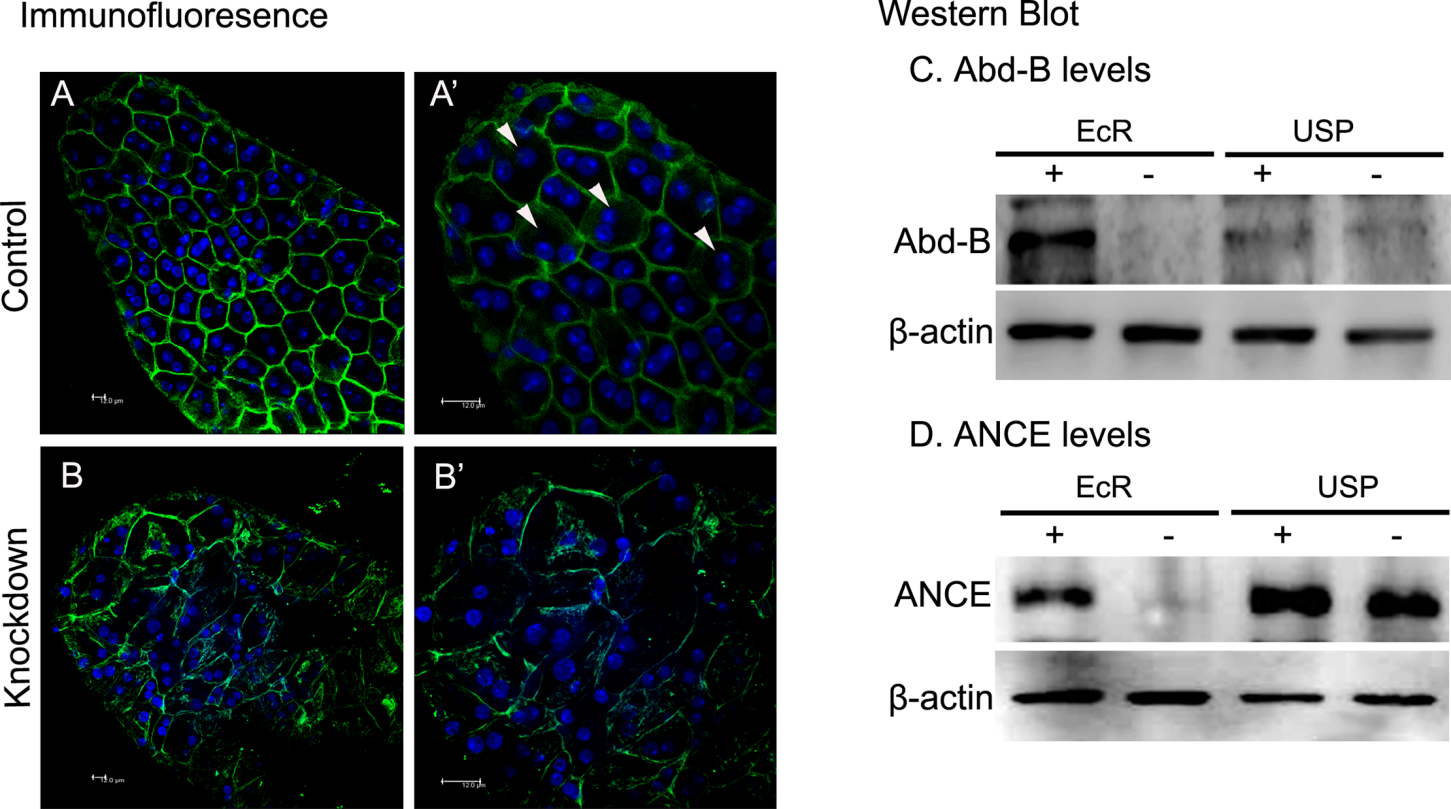
The effect of EcR knockdown on the cellular organization of the accessory glands. Immunofluorescence panels shown here are the overlay images of accessory glands immunostained with α-Spectrin antibody (marking cell membrane, Green color) and labeled with nuclear stain DAPI (blue color). In EcR control glands, several polygonally shaped binucleate cells (the main cells) and a few large and spherical binucleate cells (the secondary cells, marked with arrow) interspersed between the polygonally shaped cells were observed at 400X (Panel A) and at a higher magnification of 630X (Panel A′). However, in EcR-deficient glands, cell membranes are highly disrupted (Panel B at 400X) and the nuclear distribution is distinctly different from that of control (Panel B′ at a total magnification of 630X). To assess the effect of depletion of EcR or USP, in accessory glands,western blots were probed for the secondary cell markers, Abd-B and ANCE. The glands lacked Abd-B (Panel C,—lane under EcR in Abd-B blot) while EcR control (Panel C, + lane under EcR), USP control (Panel C, + lane under USP) or USP knockdown (Panel C, -lane under USP) had detectable levels of Abd-B. In addition, EcR knockdown glands did not contain ANCE (Panel D, -lane under EcR in ANCE blot) while the same could be detected in controls, suggesting the disruption of secondary cells due to depletion of EcR.

Each male accessory gland lobe of Drosophila has a single cellular layer comprised of morphologically distinct main cells and secondary cells with a muscle sheath underneath surrounding the lumen [27,28]. These main cells and secondary cells express different but partially overlapping sets of Acps [29]. To test if depletion of EcR affects the accessory gland structure, we compared the morphology and ultrastructure of EcR-depleted accessory glands with that of EcR control or USP control and knockdown glands. In controls (S9A & S9C Fig) and USP knockdown males (S9D Fig), the accessory glands under phase contrast microscope appeared bulged and their lumina were filled with secretions. However, accessory glands of EcR knockdown males (S9B Fig) appeared flaccid and contained little/no secretions in their lumina. Transmission electron micrographs of the accessory gland in the EcR control males contained a single layer of epithelium, with secretory cells, numerous ribosomes, endoplasmic reticulum and Golgi bodies. The lumen contained several vesicles filled with electron dense secretions (Fig 5A). In addition, the lumen was also filled with various filamentous structures signifying the secretory proteins (Fig 5A). However, accessory glands from EcR knockdown males contained disorganized endoplasmic reticulum, fewer ribosomes, and the lumen contained fewer/negligible filamentous structures and was characterized by extensive vacuolization (Fig 5B).The ultrastructure of USP control and knockdown accessory glands did not deviate from that of EcR controls and contained several filamentous structures (Fig 5C & 5D). To understand the effects of EcR depletion, if any, on the cell types (main cells and secondary cells), we immunostained the glands with antibodies against α-Spectrin (which label the cell membrane, as in Minami et al. [31]). In the EcR control, we observed several polygonally shaped binucleate cells (the main cells) and a few large and spherical binucleate cells (the secondary cells) interspersed between the polygonally shaped cells (Fig 6A and 6A′). In contrast, EcR-deficient glands lacked such cellular distinction and contained highly disrupted cell membranes with aggregated nuclei (Fig 6B and 6B′), indicating the effects of EcR depletion on the cellular organization in the male accessory glands. To substantiate the effects of EcR depletion on the cell types of accessory gland, we performed western blotting of the glands with antibodies for Abdominal-B (Abd-B), a Hox protein essential for normal development of the secondary cells [29]. Interestingly, Abd-B protein was significantly reduced /non-detectable in accessory glands from EcR knockdown males (Fig 6C,—lane under EcR in Abd-B panel), while controls had detectable Abd-B protein (Fig 6C, + lane under EcR, + and–lanes under USP in Abd-B panel) suggesting that the development/function of secondary cells might be affected. To corroborate the same, we analyzed the levels of Angiotensin I-converting enzyme (ANCE), which is expressed and associated with large vesicles of secondary cells [51]. Expectedly, controls had detectable ANCE (Fig 6D, + lane under EcR, + and–lanes under USP in ANCE panel) while the same in accessory glands from EcR knockdown males was barely detectable (Fig 6D,—lane under EcR in ANCE panel) indicating the disruption of secondary cells in EcR knockdown glands. To further characterize the effect of EcR knockdown on main and secondary cells of the accessory glands, the distinct secretory products (Acps) of these two cell types were analyzed.

**Fig 7:**
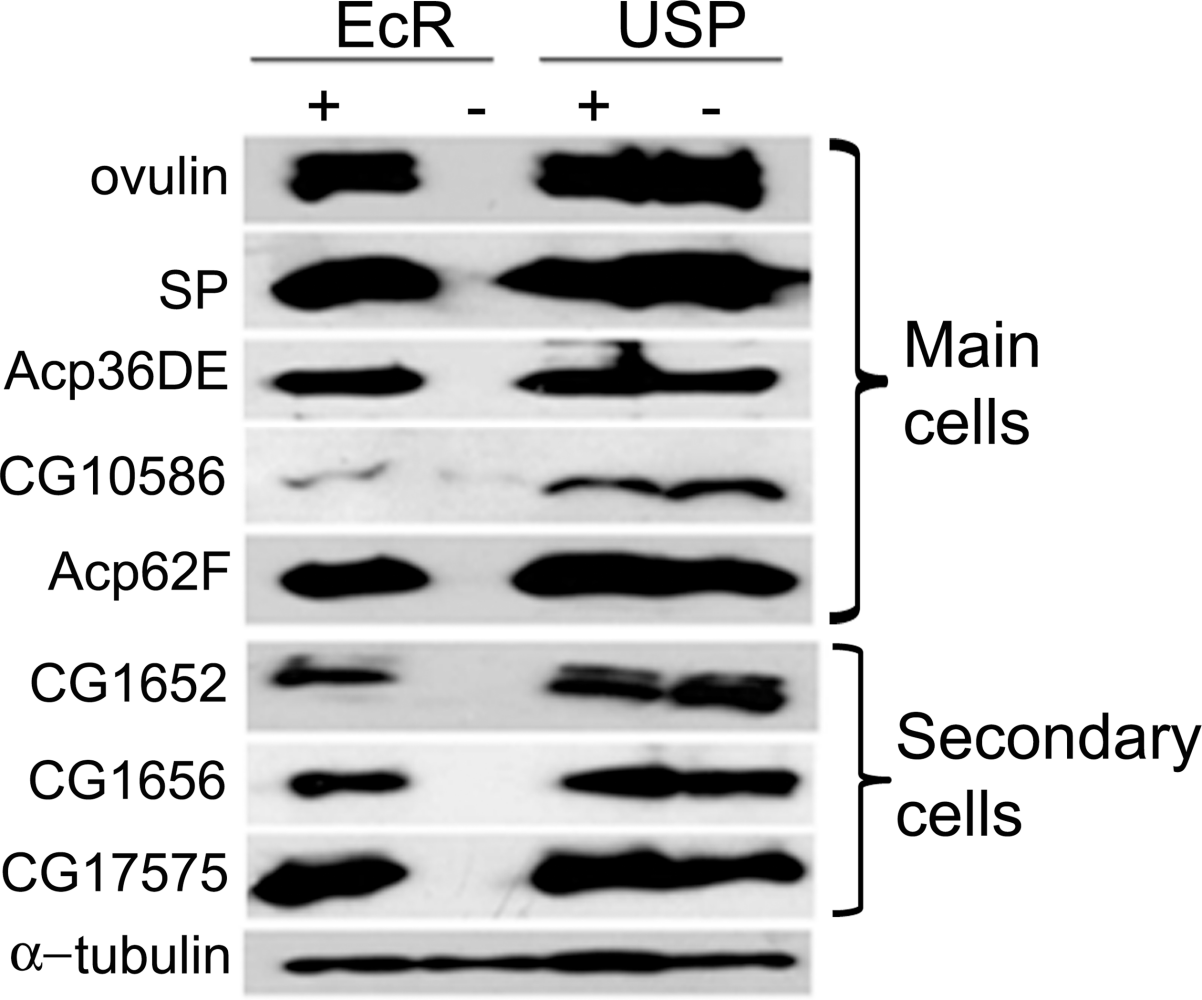
Main cell or secondary cell derived Acps in EcR or USP deficient male accessory glands. Depicted here are the western blots of accessory gland protein extracts from EcR control (+ lane under EcR), EcR knockdown (- lane under EcR), USP control (+ lane under USP) and USP knockdown (- lane under USP) males. All the Acps known to be derived from main cells (Ovulin, SP, Acp36DE, CG10586, and Acp62F) or secondary cells (CG1652, CG1656, CG17575) were detectable in EcR control, USP control and USP knockdown glands but not in EcR knockdown glands. α-tubulin was used as control for protein loading.

Of the Acps known to be associated with post-mating response in Drosophila [36,52–54], Ovulin, Sex peptide, Acp62F, Acp36DE and Seminase (CG10586) are produced by main cells while CG1652, CG1656 and CG17575 are primarily produced in the secondary cells [29]. Therefore, we analyzed the protein levels of these Acps in knockdown and control males through western blotting using anti-Acp antibodies. All the Acps (Ovulin, Sex peptide, Acp36DE, CG1652, CG1656, CG17575, CG10586, Acp62F), for which blots were probed, were detectable in samples from the accessory glands of EcR control males (Fig 7, + lane under EcR) and USP control (Fig 7, + lane under USP) or knockdown males (Fig 7,–lanes under USP) at comparable levels. Consistent with our above observation on the lack/disruption of secondary cells, accessory gland protein samples from EcR knockdown males did not have detectable CG1652, CG1656 and CG17575 levels (Fig 7,—lane under EcR). In addition, these glands also lacked the Acps (Ovulin, Sex peptide, Acp62F, Acp36DE and Seminase; Fig 7,—lane under EcR) produced in the main cells indicating that deficiency of EcR affected both main and secondary cells of the accessory gland. Consistent with this, quantitative PCR analysis in EcR-TRiP based knockdown males revealed a significant reduction of transcript levels of candidate Acps representing secondary cells (CG1652, CG1656) and main cells (CG11864) in comparison to controls (S10 Fig, *p*<0.05). The extreme vacuolization and the lack of candidate Acps typically contributed by main and secondary cells suggest that loss of EcR affects both cell types and that EcR is necessary for normal functional male accessory glands in Drosophila.

### Knockdown of EcR induces cell death in Drosophila male accessory glands

**Fig 8:**
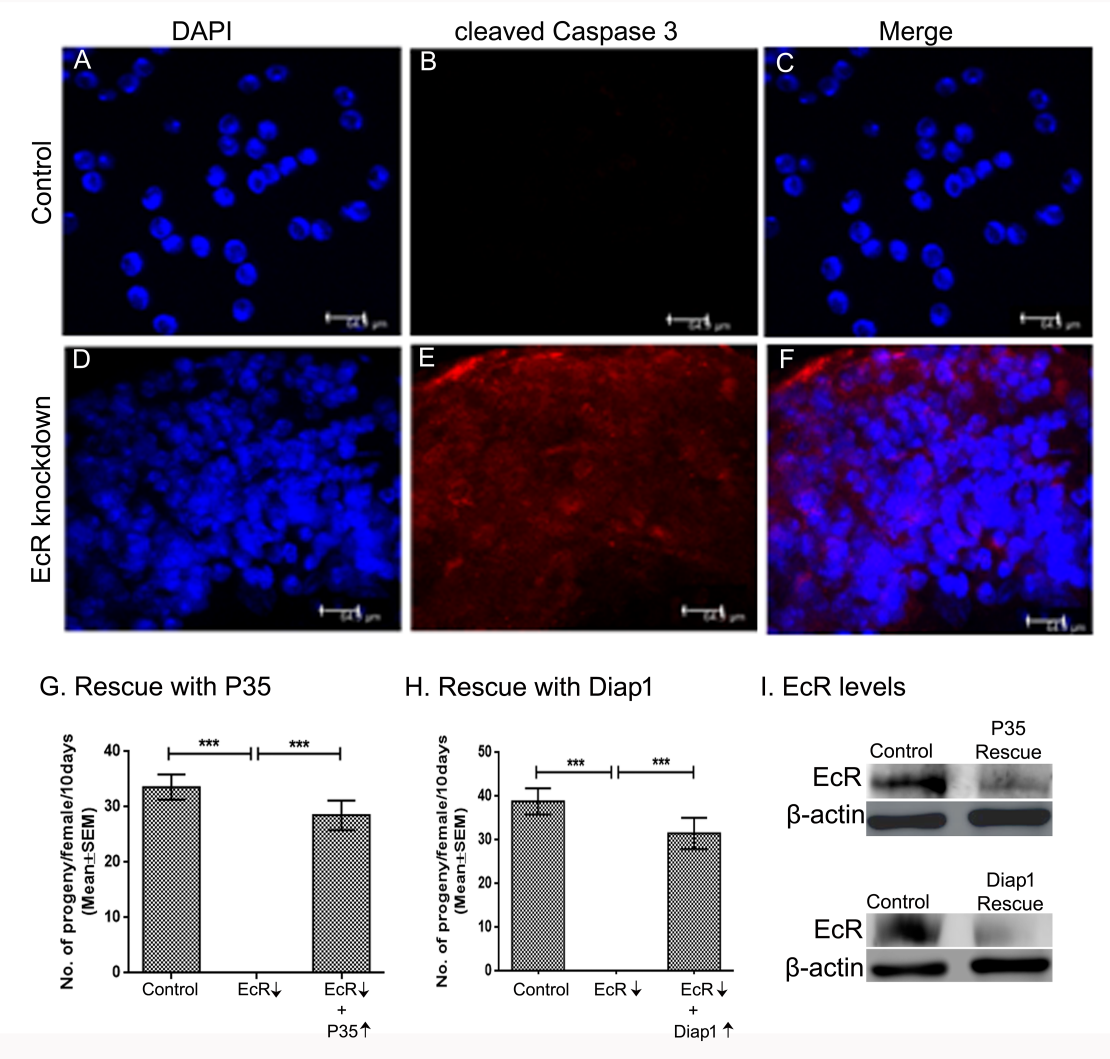
Analysis of cleaved Caspase 3 immunoreactivity in accessory glands of EcR control and knockdown males. To examine if loss of EcR leads to induction of apoptosis in accessory glands, tissues were immunostained with antibodies for cleaved Caspase 3, which react with initiator and effector caspases in Drosophila. Control tissues show well organized nuclei (Blue in color; DAPI, Panel A) and lack of detectable cleaved Caspase 3 immunoreactivity (Panel B) the overlay (Panel C) shows only the nuclei, in contrast, glands from EcR knockdown contain distorted as well as disorganized nuclei (DAPI, Panel D) and high levels of cleaved Caspase 3 immunoreactivity (Panel E). The overlay (Panel F) shows distorted nuclei (blue) and cleaved Caspase 3 labeling (red). (G) Females mated to EcR knockdown males over expressing P35 (EcR↓+P35↑) produced progeny at control levels as opposed to sterility in EcR knockdown mates (EcR↓, ***p<0.0001) indicating that over-expression of P35 rescued the fertility of EcR knockdown males. (H) EcR knockdown males overexpressing Diap1 (EcR↓+Diap1↑) were fertile as opposed to sterility in EcR knockdown mates (EcR↓, ***p<0.0001) indicating that overexpression of Diap1 rescued the fertility of EcR knockdown males. (I) Westerns blots of accessory gland proteins to confirm the knockdown status of EcR in males over expressing P35 (EcR↓+P35↑) or Diap1 (EcR↓+ Diap1↑) in EcR knockdown background. Blots were probed with anti-β-actin antibody as control for protein loading.

In Drosophila, down-regulation of EcR is known to induce cell death in prothoracic gland (PG) through apoptosis [55]. In Drosophila, the regulation and execution of steroid induced apoptosis is dependent on the interplay between anti-apoptotic gene *Drosophila inhibitor of apoptosis protein 1* (*Diap1*) and proapoptotic genes *reaper (rpr)*, *head involution defective (hid)* and *grim*, which activate a family of proteases known as caspases [56–59]. Therefore, to test if EcR depletion is leading to cell death in male accessory glands in the present study, we immunostained these glands with cleaved Caspase 3 antibody, which detects active effector caspases (Drice and Dcp-1) in apoptotic cells and the activity of Caspase-9 like initiator caspase (Dronc) in Drosophila [60]. In controls, accessory glands had binucleate cells (Fig 8A) with intact nuclei as reported above, and cleaved Caspase 3 labeling was non-detectable (Fig 8B & 8C). In contrast, EcR-deficient male accessory glands had distorted nuclei (Fig 8D) reminiscent of nuclear damage. In addition, these glands contained increased labeling of cleaved Caspase 3 (Fig 8E & 8F), suggesting apoptosis in these accessory glands. Taken together, these data indicate that EcR deficiency induces apoptosis in male accessory glands and this could explain the lack of functional products (Acps) in these glands. It is well known that inhibition of caspase activity and promotion of their degradation by Diap1 can suppress cell death [61,62]. In addition, over-expression of Baculovirus P35 is known to rescue cells from undergoing apoptosis by interfering with the cell death pathway [63]. Therefore, if the observed EcR knockdown fertility phenotype is a consequence of increased levels of apoptosis in accessory glands, one would expect the overexpression of Baculovirus P35 or the Diap1 in the EcR knockdown background to rescue the fertility. As predicted, we observed that overexpressing P35 (Fig 8G) or Diap1 (Fig 8H) in the EcR knockdown background (Fig 8I) indeed rescued the fertility of these knockdown males. Unlike the EcR depleted glands, accessory glands from EcR knockdown males overexpressing P35 or Diap1were filled with secretions and morphologically comparable to the control gland (S11 Fig). Further, females mated to EcR knockdown males overexpressing P35 (Fig 8G, *p* = 0.2260) or the anti-apoptotic Diap1 (Fig 8H, *p* = 0.2055) had fertility at levels comparable to control.

### The active receptor may be composed of EcR isoforms

In the present study, observations at the morphological, structural, transcriptional and biochemical levels demonstrate that EcR is necessary for the normal development/function of male accessory glands and that USP is not required for these functions in Drosophila. Interestingly, a similar paradigm was observed by Costantio *et al*. [64] in the expression of genes encoding glue proteins in the salivary glands of Drosophila. Together, it appears that a novel EcR based receptor that does not contain USP plays critical role(s) in regulating the gene expression in the secretory tissues (such as accessory glands and salivary glands) of Drosophila. At this juncture, it is highly intriguing how these novel receptors regulate somatic (glue protein production by salivary glands) and sex-specific functions (male accessory gland development) and thus opens up future avenues for deciphering somatic, tissue specific and sex-specific regulatory networks underlying hormonal signaling in insects.

**Fig 9:**
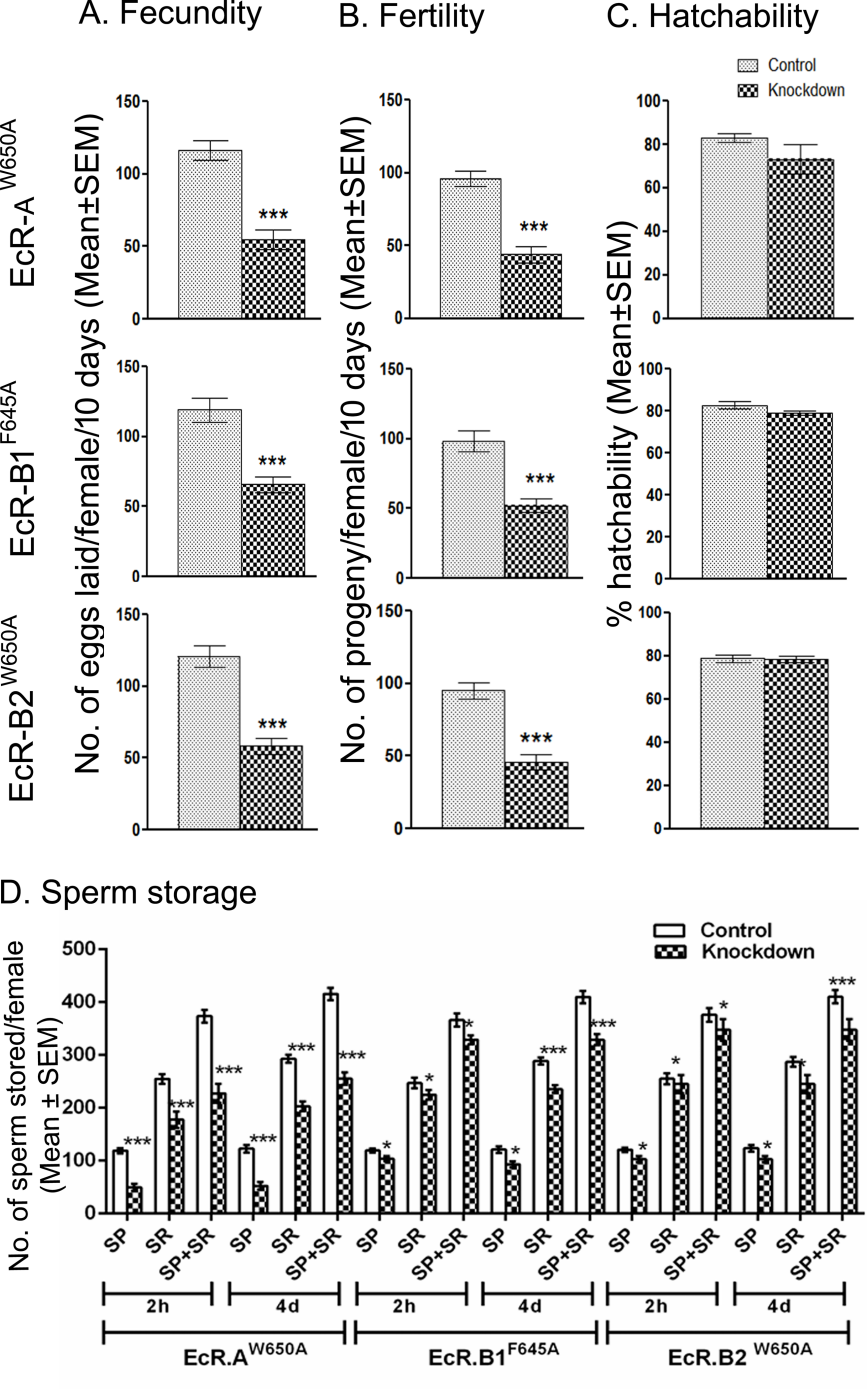
Reproductive performance of females mated to males expressing dominant negative isoforms of EcR. Panels (A), (B) & (C) represent fecundity, fertility and % hatchability, respectively of females mated to males over expressing EcR-A, EcR-B1, or EcR-B2 over a period of 10 days. Females mated to any of these three laid significantly fewer eggs and produced fewer progeny (***p <0.0001) when compared to their respective controls. However, there was no significant effect on % hatchability (*p*>0.05). (D) Sperm stored in spermathecae (SP) or seminal receptacle (SR) and total sperm in storage (Total) of females mated to males over expressing EcR-A, EcR-B1, or EcR-B2 respectively over a period of 2 hrs and 4 days ASM. Significance is ascribed as ***p<0.001; *p<0.05. Values given here are Mean±SEM involving tissues from at least 15–20 females.

**Fig 10:**
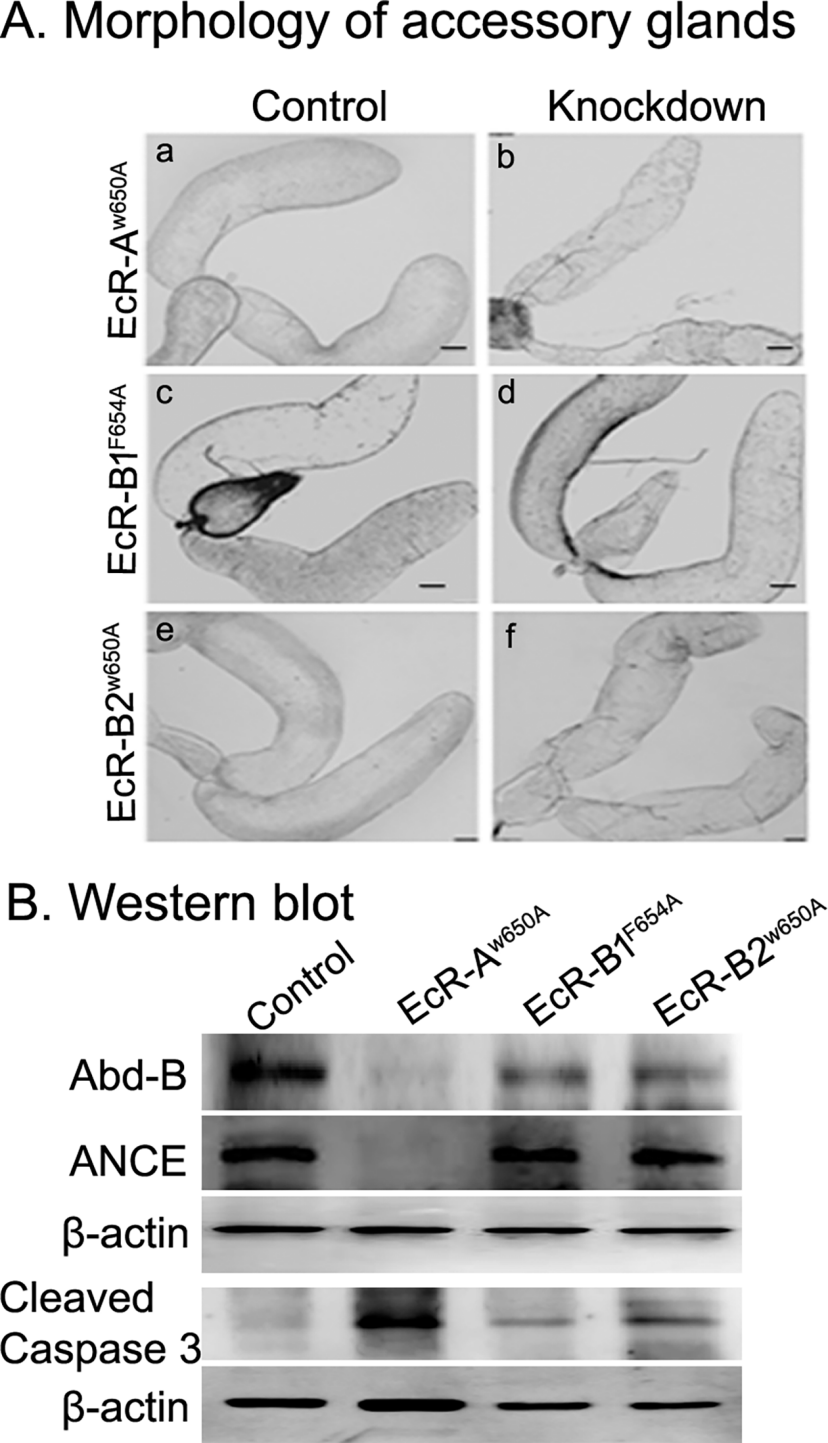
Morphology and secondary cells markers of accessory glands in males over-expressing dominant negative EcR isoforms. The morphology of accessory glands from (A) control or males over expressing (B) EcR-A, (C) EcR-B1, or (D) EcR-B2 was observed under light microscopy. Morphology of accessory glands from males over expressing EcR-B1, or EcR-B2 is comparable to their controls. However, accessory glands from EcR-A appear slightly reduced in comparison to their controls but still not as extremely reduced as those in EcR-miRNA based knockdown males. (B) Western blots of accessory gland protein extracts depicting levels of Abd-B (Abd-B panel), ANCE (ANCE panel) proteins and cleaved Caspase 3 immunoreactivity (cleaved Caspase 3 panel) in males over expressing EcR-A, EcR-B1 and EcR-B2. Blots were probed with β-actin antibodies (β-actin panels) served as controls for protein loading.

Nuclear receptors are known to function as dimers [43,65] in transmitting the hormone signal. Therefore, two possible compositions exist for the active receptor in transmitting the hormonal signal to male accessory glands in Drosophila. First, EcR may complex with another member of the nuclear receptor superfamily in the accessory gland to transmit the hormonal signal. In this case, one would expect that silencing of such a nuclear receptor partner in the accessory glands leads to a phenotype of reduced fertility. However, the lack of significant fertility reduction in the present study with the RNAi of nuclear receptors other than EcR argues against the involvement of another member of nuclear receptor superfamily as a part of the active receptor at this tissue level. A second possibility is that EcR may form homodimers involving its isoforms. EcR encodes three isoforms, namely EcR-A, EcR-B1 and EcR-B2, with the conserved C-terminal ligand binding domain having transcriptional activation function and a variable N-terminal A/B domain [66]. To test the involvement of EcR isoforms, we analyzed reproductive parameters (fecundity, fertility, hatchability and sperm storage) and certain accessory gland parameters [morphology, secondary cell markers (Abd-B and ANCE) & apoptosis (cleaved Caspase 3)] in males overexpressing the dominant negative forms of EcR-A (*EcR-A*^*W650A*^), EcR-B1 (*EcR-B1*^*F645A*^) or EcR-B2 (*EcR-B2*^*W650A*^), in an accessory gland-specific manner. Interestingly, females mated to males overexpressing any of these dominant negative forms laid fewer eggs (Fig 9A, *p*<0.0001) and produced fewer progeny (Fig 9B, *p*<0.0001) when compared to their controls while hatchability (Fig 9C, *p*>0.05) paralleled that of controls. Similarly, these mated females had significantly fewer sperm in their sperm storage organs, namely, spermathecae and seminal receptacle at 2h and 4 days ASM when compared to their controls. It was observed that although there is a significant difference in sperm storage in mates of all the three dominant negative forms, storage was more severely affected in females mated to males expressing EcR-A than those of EcR-B1 or EcR-B2 (Fig 9D). Further, in comparison to controls (Fig 10A-a, 10A-c & 10A-e), the reduction in the size of the accessory glands was quite noticeable with the overexpression of EcR-A (Fig 10A-b) but not with the overexpression of EcR-B1 (Fig 10A-d) or EcR-B2 (Fig 10A-f). In addition, both Abd-B and ANCE (the secondary cell specific candidates) were drastically reduced in EcR-A^*W650A*^ accessory glands (Fig 10B). Accessory glands from EcR-B1^*F645A*^ or EcR-B2^*W650A*^ had marginally reduced Abd-B levels when compared to that in control while ANCE levels were comparable to control (Fig 10B). Similarly, cleaved Caspase 3 signals were detectable in all three as opposed to the control but the signal of cleaved Caspase 3 was relatively higher in EcR-A^*W650A*^ glands (Fig 10B). Consistent with this, cleaved Caspase 3 labeling was detectable in glands from all three isoforms but the intensity of labeling was higher (yet lower than that in the EcR knockdown) in EcR-A^*W650A*^ than in EcR-B1^*F645A*^ or EcR-B2^*W650A*^ (S12 Fig). These observations may point to a major role for EcR-A in the development of male accessory glands. However, at this juncture, it is important to note that individual overexpression of these isoforms does reduce male fertility, sperm storage and induce apoptosis in the gland but not to the same intensity as knockdown of all the isoforms (through EcR-miRNA or EcR-TRIP). In addition, none of the glands from EcR-A^*W650A*^, EcR-B1^*F645A*^ or EcR-B2^*W650A*^ manifested the abnormal nuclear distribution/aggregation observed in glands (EcR-miRNA) lacking all three isoforms (S12 Fig). Therefore, rather than one particular isoform, the active receptor may be a homodimer, probably involving all the isoforms of EcR. However, further studies are required to delineate the specific role each EcR isoform has in regulating male accessory gland development. Nonetheless, the above findings suggest that EcR, along with its all isoforms, is essential for male fertility in Drosophila.

In Drosophila, male accessory glands are derived from the male genital disc under the influence of sex-specific regulatory network involving *doublesex* (*dsx*), *Drop* (*Dr*) and *AP-2* and Fibroblast Growth Factor (FGF) signaling [26,67]. Subsequent cell proliferation and functional differentiation of accessory glands require Drosophila homeodomain transcription factor *prd* [24], homeodomain transcriptional repressor *defective proventriculus* (*dve*) [31] and Hox gene *Abdominal-B (Abd-B)* [29]. In the present study, depletion of EcR in a *prd*-GAL4 dependent manner resulted in defective accessory glands that also lacked detectable Abd-B. However, the same EcR dsRNA when driven through an *ovulin* (an Acp gene) promoter-based GAL4 did not result in a detectable effect on fertility (S13 Fig). These data suggest that EcR functions downstream of *prd* and might act early, before the transcriptional activation of genes encoding Acps in the male accessory gland.

To conclude, the critical requirement for EcR in Drosophila male fertility has potential applications in designing pest control strategies and in toxicology. Our study projects EcR as a potential target for biotechnological approaches of sterile male technique [68–70], which is extensively used for controlling agricultural pests and disease vectors. From a toxicological perspective, the study offers a molecular handle with which to gain insights to environmental chemical (xenobiotic) interference with endocrine function in insect pollinators and other economically important insects.

## Materials & methods

### Flies

Transgenic *D*. *melanogaster* lines carrying miRNA (UAS-miRNA) based constructs for 18 nuclear/hormone receptors (EcR, ERR, DHR39, DSF, USP, DHR78, DHR51, DHR96, SVP, E78, DHR38, DHR83, FTZ-F1, EiP75, DHR3, NHF4, TLL, and DHR4) were from Lin *et al*.[71]. Further, TRiP lines for EcR (BL58286, TRiP.HMJ22371), Met (BL26205, TRiP.JF02103), Jhe (BL57826, TRiP.HMJ21834), USP (BL36729, TRiP.HMS01620), *prd*-GAL4/TM3 *Sb* (BL1947), G-TRACE line [BL2880, 72], dominant negative lines for EcR-A (BL9451, UAS-*EcRA*.W650A), EcR-B1 (BL6869, UAS-*EcR*.*B1*-ΔC655.F645A) and EcR-B2 (BL9449, UAS-*EcR*.*B2*.W650A), overexpression lines of P35 (BL5073, UAS-*P35*) and Diap1 (BL6657, UAS-*diap1*), were obtained from Bloomington Drosophila Stock Centre, USA. An additional USP-RNAi line (VDRC 4380) was obtained from Vienna Drosophila Resource Center, Austria. The two EcR or three USP knockdown lines used in the present study carry different constructs and target different regions of EcR or USP transcripts, respectively. In addition, two independent prediction programs namely, dscheck [73] or online OTE (off-target effect) prediction tool of DRSC (www.flyrnai.org) did not yield any potential off-targets for the concerned dsRNA generating sequences. For assays involving fertility and sperm storage, flies (particularly females) from wild type *D*. *melanogaster* (Oregon-R) were used. ProtamineB-EGFP/ProtamineB-EGFP; *prd*-GAL4/TM3 *Sb* flies were generated through crosses between GAL4 driver and ProtamineB-EGFP; TM3/TM6 flies [74] to study the effect of hormone receptor knockdown on sperm production, transfer and storage. All flies were maintained on standard maize-sugar Drosophila food medium [75] supplemented with yeast (12:12h light-dark cycle at 22±2°C).

### Generation of control and knockdown males

By crossing UAS-*HR-miRNA* males with *prd*-GAL4/TM3 *Sb* females, males knockdown (*prd*-GAL4;UAS-*HR-miRNA*) for hormone receptors, individually, in the accessory glands and their genetically matched controls (UAS-*HR-miRNA*; TM3 *Sb*) were generated. Subsequently, 3–5 days old unmated knockdown or control males, isolated within 3h of their eclosion, were crossed to 3–5 days old Oregon-R (wild type strain of *D*. *melanogaster)* virgin female for different assays as described below.

### Fertility, fecundity & hatchability assays

Males were allowed to mate with Oregon-R virgin females in pairs and then were discarded after mating. Mated females were allowed to lay eggs for ten days with the change to fresh food vials at the interval of 24h for fecundity (no. of eggs laid)/fertility/hatchability assays or on three days basis for standalone fertility assays. Females were discarded after 10 days ASM (after the start of mating) and the resultant progeny were counted to determine fertility [30]. The proportion of eggs that reached adulthood was considered as percent hatchability. The difference in fecundity, fertility and hatchability between females mated to knockdown or control males were analyzed through Mann-Whitney U test. Experiments were repeated three times with at least 15–20 replicates for each group. In all cases, genetically matched siblings were used as controls.

### Sperm production, transfer and storage

To examine sperm, males expressing miRNA or dominant negative forms were crossed with ProtamineB-EGFP; *prd*-GAL4/TM3 *Sb* females for the generation of knockdown and control males with GFP-tagged sperm. For sperm production, the male reproductive tract was dissected in PBS from 3–5 days old control/knockdown males (minimum 5 replicates for each group) and was observed under a confocal microscope (Leica TCS SPE, Leica, Germany) for the presence of mature sperm in seminal vesicles. Transfer and storage of sperms in females mated to control or knockdown males were analyzed at 2h ASM (after the start of mating) as reported in Neubaum and Wolfner [54]. A minimum of five replicates of control and knockdown males was pair mated with wild type virgin females. Males were discarded after mating. Reproductive tracts dissected out of mated females at 2h ASM in PBS, were placed in a drop of mounting medium on slides and coverslips were sealed with nail polish. GFP-labeled sperm in the male/female reproductive tracts were visualized under a confocal microscope at a total magnification of 63X (Leica TCS SPE, Germany). A minimum of 10 tissues from control and knockdowns was analyzed for the pattern of sperm production/transfer/storage. Further, sperm stored in both seminal receptacle and spermathecae were counted under a fluorescent microscope (Leica DMBL, Germany) following the protocol of Mueller *et al* [76]. Sperm stored in each storage organ was counted twice and the repeatability index for the sperm counts was 95% (5 replicates per group). Significant differences, if any, in the number of sperm stored by control and knockdown mates were analyzed through the Mann-Whitney U test.

### Phase contrast microscopy

Observation of accessory gland morphology was done under a phase contrast microscope. Accessory glands dissected from 3–5 days old control or knockdown males were mounted on a slide with a droplet of physiological saline and sealed with a coverslip. These tissues were observed under an inverted microscope at a total magnification of 100X and 200X (Nikon Japan). A minimum of five tissues was analyzed per group (control or knockdown).

### Transmission electron microscopy

The ultrastructure of accessory glands from knockdown and control males were analyzed through Transmission Electron Microscopy. Briefly, accessory glands from 3–5 days old males were dissected in PBS and were processed through conventional procedure of tissue fixation used for electron microscopy including 2% Paraformaldehyde, 2% Glutaraldehyde (prepared in 0.1M Sodium cacodylate buffer) treatment followed by three times washing in 0.1M Sodium cacodylate (5 min each) & 1% Osmium tetraoxide treatment (2 h). Subsequently, tissues were dehydrated in 15%, 30%, 60%, & 100% acetone (20 min each). These dehydrated tissues were embedded in a low viscosity epoxy resin araldite -502 (Ted Pella, Inc., Redding, CA, USA) medium to prepare blocks. Semi and ultrathin sections were cut using a Leica EM UC7 ultramicrotome (Ultra Cut UCT ultramicrotome, Leica, Vienna, Austria). The sections were stained by uranyl acetate and lead citrate and structural patterns were examined on a FEI Tecnai G2 spirit TWIN transmission electron microscope. A minimum of 10–15 pairs of accessory glands per group was taken and the experiments were repeated three times.

### Immunostaining

The accessory glands from the male reproductive tract were dissected in 0.7% saline and placed in 1X PBST (1X PBS with 0.3% Triton-X) until the end of all the dissections. Subsequently, PBST was replaced with 4% PFA (Paraformaldehyde in 1X PBS), and the tissues were incubated for 1h. The tissues were then washed with 0.3% PBST thrice, for 15 min each. The blocking solution (4% BSA in 0.1% PBST) was added, and tissues were incubated for 30 min. The primary antibodies for cleaved Caspase 3 (Asp175, 1:100, primary host-rabbit, cell signaling technology, USA) or α-Spectrin (3A9, 1:100, primary host-mouse, DSHB, USA) were added, and the tissues were incubated overnight at 4°C. Tissues were then washed thrice, for 15 min each with 0.3% PBST, and incubated in Alexa Fluor® 568 Goat Anti-Rabbit (Life technologies, USA) or Alexa Fluor® 488 Goat Anti-Mouse (Life technologies, USA), respectively, diluted in 0.1% PBST at 1:200 at room temperature for 2 h. Tissues were washed thrice, for 15 min each with 0.3% PBST and mounted on slides using mounting media containing DAPI (vectashield, vector laboratories, USA). The images were taken under a confocal microscope (TCS SPE, Leica, Germany), on DAPI (excitation wavelength 340-380nm), FITC (excitation wavelength 450-490nm) or TRITC (excitation wavelength 578-603nm) settings, depending upon the Alexa fluors used. Three independent batches with five replicates (accessory gland pairs)/batch were used for each group.

### Western blotting

Protein samples were prepared from accessory glands dissected out of 3–5 days old knockdown or control males and were probed through western blotting. Briefly, four pairs of accessory glands were homogenized in 20μl of 2X SDS sample buffer [77], boiled for five minutes and were loaded and resolved on 7.5–15% gradient SDS-polyacrylamide gel for detection of Acps, ANCE, Abd-B or cleaved Caspase 3 while proteins were loaded onto 7.5% SDS-polyacrylamide gels for detection of EcR and USP. After SDS-PAGE, proteins were transferred to a PVDF membrane (Millipore, Merck Life Science, Bangalore, India) using semi-dry transfer system (Amersham Biosciences, USA). The blots were probed with antibodies against EcR, [primary antibody: Anti-Ecdysone receptor antibody (ab35264, Abcam, USA) at a dilution of 1:1000; Secondary antibody: peroxidase affinipure rabbit anti-sheep at a dilution of 1:2000 (Jackson, USA)], USP [primary antibody: Anti-USP antibody (ab106341, Abcam, USA) at dilution 1;1000; Secondary antibody: Peroxidase–Affinipure Goat anti-rabbit secondary antibody at a dilution of 1:2000 (Jackson, USA)], candidate seminal fluid proteins {Anti-Acp antibodies for Ovulin, SP, Acp36DE, CG1652, CG1656, CG17575, CG10586, Acp62F; Ravi Ram *et al*. [77]}, Abd-B [anti-Abd-B (1A2E9, DSHB, USA) at a dilution 1:750; Secondary antibody: Peroxidase–Affinipure Goat anti-mouse secondary antibody at a dilution of 1:1500 (Jackson, USA)], anti-ANCE [1:1000 dilution, [51]; Secondary antibody: Peroxidase–Affinipure Goat anti-rabbit secondary antibody at a dilution of 1:2000 (Jackson, USA)] or cleaved Caspase 3 [Asp175, 1:1000, Cell Signaling Technology, USA; Secondary antibody: Peroxidase–Affinipure Goat anti-mouse secondary antibody at a dilution of 1:2000 (Jackson, USA)]. As a control for the loading, the blots were probed with anti-α-tubulin antibody [75] or anti-β-actin antibody (Sigma, USA). The blots were developed with chemiluminescent agent (SuperSignal West Femto, Thermo-Fisher, USA). An equal volume of the enhancer (luminol) was mixed with stable peroxide buffer (150μl each) and spread uniformly over the blot. The patterns were documented using Versa-doc Imaging system (Bio-Rad, USA). A minimum of two independent blots was used for antibody probing.

## Supporting information

S1 Fig Confirmation and determination of the extent of knockdown of hormone receptors through quantitative real time PCR. The levels of targeted hormone receptor transcripts were significantly reduced by 5–30 folds in miRNA based knockdown driven by *prd*-GAL4 (Knockdown, *p<0.05, ** p<0.001, ***p<0.0001) when compared to those in controls (Control). The Δct values were determined through normalization against *Act-5c*, which was used as an internal control for the quality of the template.
(PDF)

S2 Fig Analysis of *prd*-GAL4 mediated expression in different larval and adult tissues of Drosophila. To confirm the tissue specificity of *prd*-GAL4 expression, we crossed the *prd*-GAL4 to G-TRACE line carrying fluorescent protein reporters for both real-time expression of GAL4 (RFP) and transient expression of GAL4 during development (enhanced GFP (EGFP)). We analyzed the brain ganglia, genital disc as well as gonads from male larvae and testes as well as accessory glands (tissues in blue, stained with DAPI) from adult males generated from the above cross for RFP or EGFP signals under a confocal microscope. We detected RFP and EGFP signals only in adult male accessory glands but not in larval brain ganglia, genital disc, gonads and adult testes, indicating the accessory gland specificity of *prd*-GAL4 driver.
(PDF)

S3 Fig Analysis of fertility and knockdown levels of EcR-TRiP males. To rule out the influence of strain background on the observed effects of EcR-miRNA, EcR was knocked down in a similar manner but with different RNAi construct (EcR-TRiP). Females mated to *prd*-GAL4; EcR-TRiP knockdown males (Panel A, Knockdown) produced significantly fewer progeny (Panel A, ***p = 0.0007; N = 15–20) when compared to their genetically matched sibling controls (Panel A, Control). To determine the extent of knockdown of EcR when EcR-TRiP is driven through *prd*-GAL4, proteins from male accessory glands of control (*Sb*; UAS-*EcR-TRiP*) and knockdown (*prd*-GAL4; UAS-*EcR-TRiP*) males were isolated and western blotting was performed with anti-EcR, anti-USP antibodies (Panel B). Protein samples from knockdown males had detectable levels of EcR but yet significantly reduced when compared to control (EcR panel). However, USP levels were comparable between control and knockdown samples (USP panel). Blots were probed with α-tubulin as a control for protein loading in control and knockdown.
(PDF)

S4 Fig The levels of USP in accessory glands from control and USP-miRNA knockdown males. In western blots, the extent of USP knockdown was quantified by running serial dilutions to the level of 50% (lane 50%),75% (lane 75%) and 100% (lane 100%), of accessory gland proteins from ten USP control males in parallel with accessory gland extracts equivalent to ten USP knockdown males (lane USP-miRNA). Three independent sets of samples were analyzed to confirm the level of knockdown. Blots probed with β-actin suggest the extent of protein loading in control and knockdown.
(PDF)

S5 Fig Fertility of females mated to USP-TRiP or USP-RNAi knockdown males. To confirm that the depletion of USP has no impact of male fertility, USP was knocked down in a similar manner but with different RNAi constructs (USP-TRiP and USP-RNAi). The number of progeny produced over a period of 10 days by females to prd-GAL4; USP-TRiP knockdown males (*p* = 0.7051; N = 15–20) and those mated to *prd*-GAL4; USP-RNAi knockdown males (*p* = 0.4711; N = 15–20) was comparable to their genetically matched controls (Control).
(PDF)

S6 Fig Fertility of females mated to EcR and USPdouble knockdown males. We hypothesized that if USP were to be involved in male fertility, depletion of USP in EcR-TRiP background should further enhance the fertility phenotype of EcR-TRiP. To test if this is the case, we generated a double knockdown of USP as well as EcR by driving the expression of both USP-miRNA and EcR-TRiP (EcR-TRiP+USP-miRNA) through *prd*-GAL4 and analyzed the fertility of females mated to these males in comparison to those mated to EcR-TRiP knockdown males. In both groups, females mated to knockdown males (Knockdown) produced fewer progeny when compared to those mated to their genetically matched sibling controls (***p<0.0007). However, the number of progeny produced over a period of 10 days by females mated to USP and EcR double knockdown males was comparable to that of EcR-TRiP knockdown mates (*p* = 0.2589). Values represented here are Mean±SEM involving at least 15–20 mated females per group and assays were repeated at least twice. The bars having same letter are statistically non-significant (control *Vs* control: *p* = 0.5493; single knockdown *Vs* double knockdown: *p* = 0.2589) and significantly differ from those with a different letter (Control *Vs* knockdown: ***p = 0.0007).
(PDF)

S7 Fig Reproductive performance of EcR-TRiP control and knockdown males. To assess the reproductive performance, we mated 3-5days old virgin females with control or knockdown males and assessed the number of eggs laid (A, Fecundity), number of progeny produced (B, Fertility) by mated females and the proportion of eggs reaching adulthood (% hatchability) over a period of 10 days ASM. Females mated to EcR-TRiP knockdown males laid significantly fewer eggs (*p = 0.03; N = 10–15). Similarly, females mated to knockdown males produced significantly fewer progeny (***p = 0.0007; N = 10–15) when compared to control. This reduction in progeny production is a consequence of reduced egg laying as well as significantly reduced hatchability of these laid eggs (**p = 0.002; N = 10–15) when compared to those of controls.
(PDF)

S8 Fig Lack of sperm in storage organs (seminal receptacle and spermathecae) of female mated to EcR knockdown male. Panels A-D show sperm storage (green) at 2h ASM in seminal receptacle of females mated to EcR control (A), knockdown (B), USP control (C) or knockdown (D). Panel E shows the number of sperm stored in seminal receptacle (SR), Spermathecae (SP) and the total sperm in storage of females mated to EcR or USP knockdown males in comparison to sperm storage levels in controls. EcR control, USP control and knockdown had comparable number of sperm in storage while EcR knockdown mates were devoid of sperm in their sperm storage organs (***p< 0.0001, N = 5).
(PDF)

S9 Fig Morphology of male accessory glands from EcR/USP control or knockdown males. Accessory glands from 3–5 days old control and knockdown (EcR or USP) males were dissected out in normal saline (10 replicates for each group) and observed under a phase contrast microscope. In EcR control (Panel A), accessory gland appeared bulged and filled with secretions. In the EcR knockdown (Panel B), accessory gland was reduced and flaccid. Panels C and D represent accessory glands from USP control and knockdown, respectively, and both are filled with secretions as in EcR controls. Images were taken at 200X under an inverted microscope.
(PDF)

S10 Fig Mis-regulation of certain genes encoding seminal proteins in EcR knockdown (EcR-TRiP) males. Analysis of transcript levels through qPCR revealed significant down-regulation (*p<0.05) of secondary cell derived Acps (CG1652, CG1656) and main cell derived Acp (CG11864) in accessory glands from EcR-TRiP knockdown males The transcript levels of *Acp36DE*, *Ovulin* (CG8982), *Acp62F* (CG1262), *Seminase* (CG10586) and *SP* (CG17673) did not differ from controls. The ΔCt values were determined through normalization against RPL32, which was used as an internal control for the quality of the template. Experiment was carried out thrice for each transcript. (*p = 0.02, **p = 0.007).
(PDF)

S11 Fig Morphology of male accessory glands from EcR knockdown males after rescue with overexpression of P35 and Diap1. Accessory glands from 3–5 days old control and knockdown (EcR or USP) males were dissected out in normal saline (10 replicates for each group) and observed under a phase contrast microscope. In the EcR control (Panel A), the accessory gland appeared bloated and filled with secretions. In the EcR knockdown (Panel B), the accessory gland was highly reduced and the lumen appeared empty. Accessory glands from EcR knockdown males over expressing P35 (Panel C) or Diap1 (Panel D) were filled with secretions as in EcR controls. Images were taken at 100X under an inverted microscope.
(PDF)

S12 Fig Analysis of cleaved Caspase 3 immunoreactivity in accessory glands of males over expressing dominant negative EcR isoforms and their controls. To examine if inhibition of any of the EcR isoforms leads to induction of apoptosis in accessory glands, tissues from EcR-A^*W650A*^, EcR-B1^F645A^, EcRB-2^*W650A*^ were immunostained with antibodies for cleaved Caspase 3 and were compared with the tissues from the control (Control) and EcR-miRNA (EcR knockdown) immunostained in parallel. The DAPI panels show the nuclei (Blue in color; DAPI) and cleaved Caspase 3 panels represent the immunoreactivity in Red color (cleaved Caspase 3) while the overlay shows the nuclei and cleaved Caspase 3 immunoreactivity (Overlay).
(PDF)

S13 Fig Fertility of females mated to knockdown males generated by driving RNAi through *ovulin*-GAL4. To test if EcR activity is required at an early or late stages of accessory gland development, UAS-*EcR-miRNA* or UAS-*USP-miRNA* were driven under the control of *ovulin* (An Acp gene) promoter based GAL4, which expresses specifically in the accessory glands at the late pupal stage after the formation of accessory glands. We did not detect significant reduction due to ovulin promoter driven EcR knockdown and the fertility of EcR knockdown mates were comparable to that of USP knockdown mates (*p>*0.1).
(PDF)
