# Correction: Functional male accessory glands and fertility in Drosophila require novel ecdysone receptor

**DOI:** 10.1371/journal.pgen.1006893

**Published:** 2017-07-12

**Authors:** Vandana Sharma, Anuj K. Pandey, Ajay Kumar, Snigdha Misra, Himanshu P. K. Gupta, Snigdha Gupta, Anshuman Singh, Norene A. Buehner, Kristipati Ravi Ram

There is an error in reference 71. The correct reference is:

71. Lin S, Huang Y, Lee T (2009) Nuclear Receptor Unfulfilled Regulates Axonal Guidance and Cell Identity of *Drosophila* Mushroom Body Neurons. PLoS ONE 4(12): e8392. https://doi.org/10.1371/journal.pone.0008392

## References

[1] Sharma V, Pandey AK, Kumar A, Misra S, Gupta HPK, Gupta S, et al (2017) Functional male accessory glands and fertility in Drosophila require novel ecdysone receptor. PLoS Genet 13(5): e1006788 doi:10.1371/journal.pgen.1006788 28493870 10.1371/journal.pgen.1006788 PMC5444863

