# Correction: Functional male accessory glands and fertility in Drosophila require novel ecdysone receptor

**DOI:** 10.1371/journal.pgen.1007524

**Published:** 2018-07-13

**Authors:** Vandana Sharma, Anuj K. Pandey, Ajay Kumar, Snigdha Misra, Himanshu P. K. Gupta, Snigdha Gupta, Anshuman Singh, Norene A. Buehner, Kristipati Ravi Ram

Fig 4 is incorrect. The authors have provided a corrected version here.

**Fig 4:**
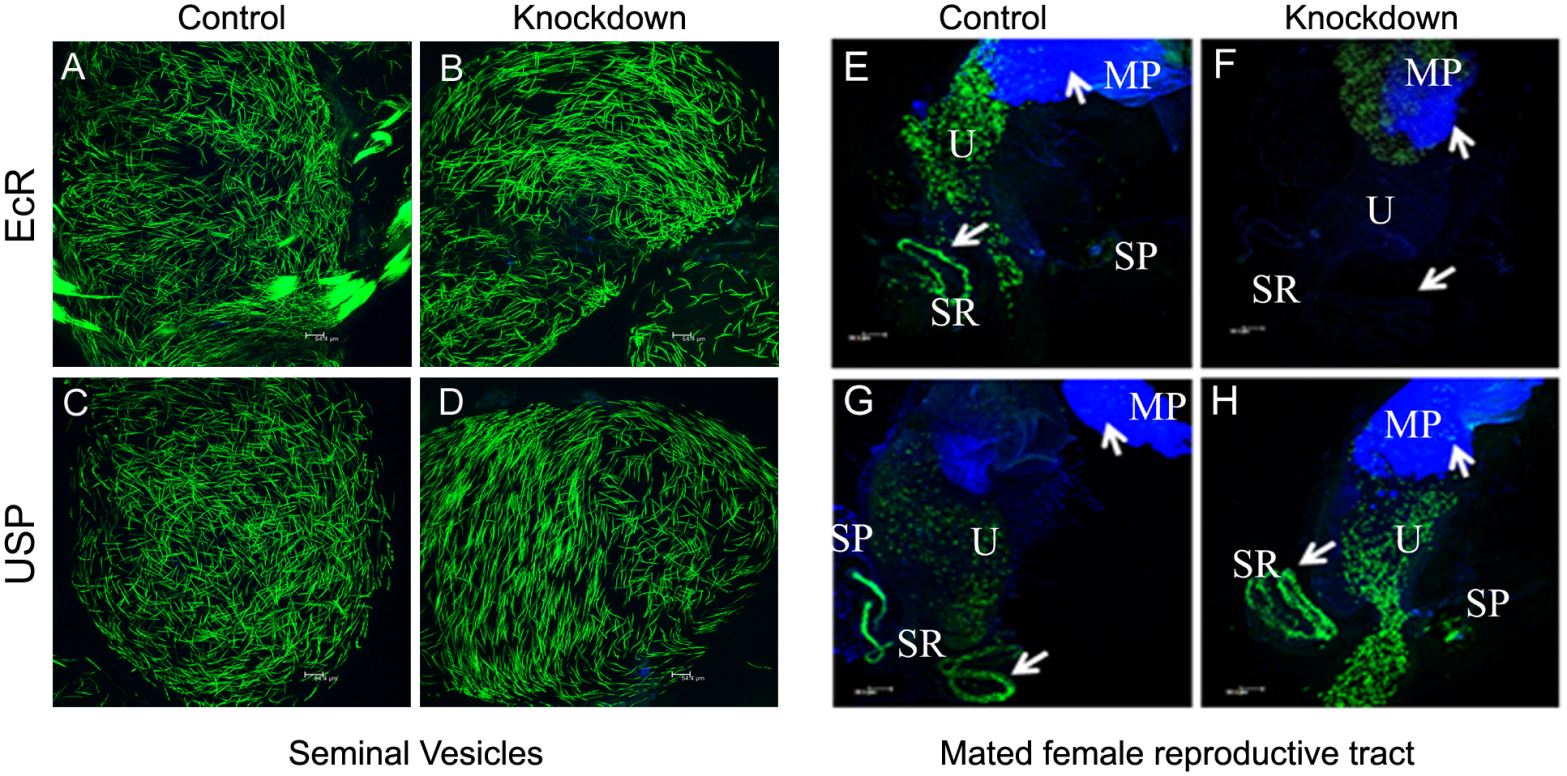
Analysis of sperm production in EcR knockdown males and their fate in mated females. To evaluate the effect of EcR knockdown (EcR-miRNA) on sperm, we first generated control and knockdown males that express GFP labeled sperm (ProtamineB-EGFP). Subsequently, observation of seminal vesicles from these males under a confocal microscope revealed comparable levels of GFP tagged sperm (green) in both controls (Panels A&C) as well as knockdown (EcR, Panel B or USP, Panel D) males, suggesting that sperm production is normal in these males. To test if these males are able to transfer sperm to females during mating, males knockdown for EcR or USP in their accessory glands were allowed to mate with Oregon-R virgin females. At 2h ASM, reproductive tracts from mated females were isolated and observed under confocal microscope for the presence of GFP-labeled sperm. Reproductive tracts from females mated to (E) EcR control, (G) USP control or (H) knockdown males contained mating plug (MP, blue) in the uteri (U) and GFP-labeled sperm (green) in the uteri as well as sperm storage organs, namely seminal receptacle (SR) and spermathecae (SP). However, reproductive tracts of females mated to EcR knockdown males (F) contained mating plugs but had sperm only in the uterus but not in SR and SP. These observations suggest that knockdown of EcR or USP has no detectable effect on sperm transfer but the sperm transferred by EcR knockdown males fail to move towards sperm storage organs and are not stored at levels comparable to those in controls.
