# Supplementary figures and images for: Functional male accessory glands and fertility in Drosophila require novel ecdysone receptor
Source: PLoS Genet. 2017 May 11;13(5):e1006788. doi: 10.1371/journal.pgen.1006788 (PMC5444863; doi:10.1371/journal.pgen.1006788)

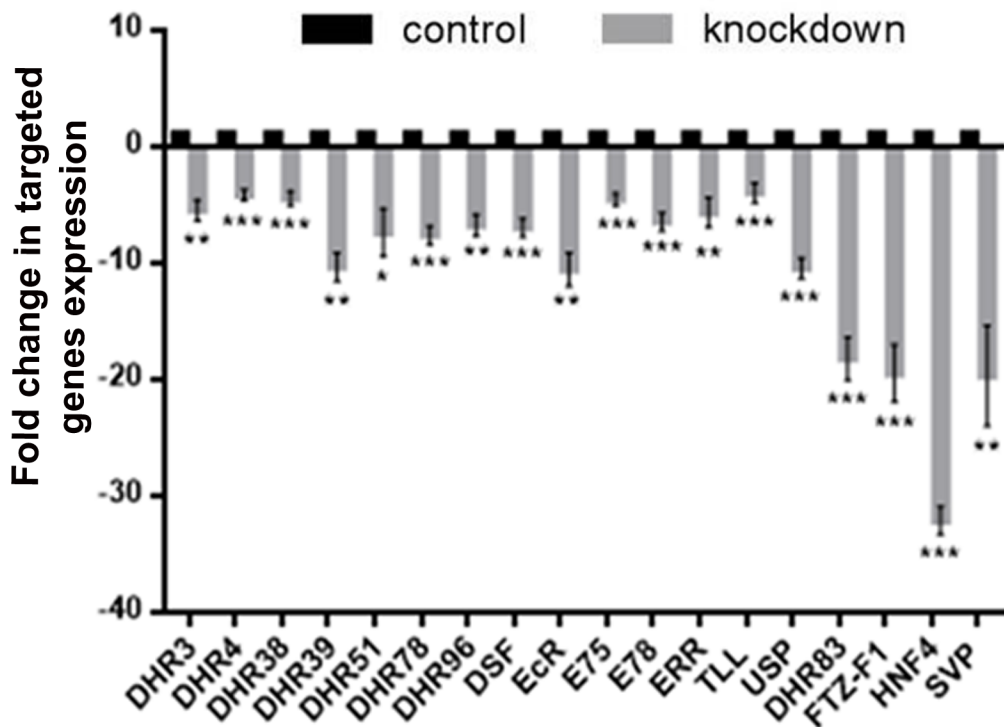

Supplement: S1 Fig — The levels of targeted hormone receptor transcripts were significantly reduced by 5–30 folds in miRNA based knockdown driven by prd-GAL4 (Knockdown, *p<0.05, ** p<0.001, ***p<0.0001) when compared to those in controls (Control). The Δct values were determined through normalization against Act-5c, which was used as an internal control for the quality of the template. (PDF) [file pgen.1006788.s001.pdf]

A.

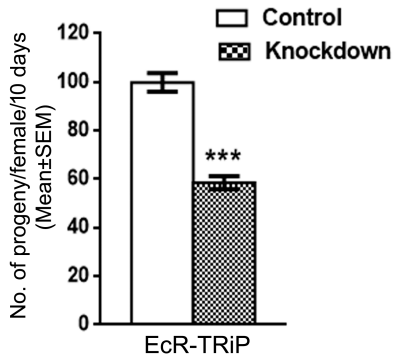

B.

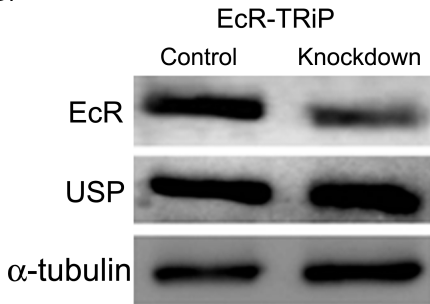

Supplement: S3 Fig — To rule out the influence of strain background on the observed effects of EcR-miRNA, EcR was knocked down in a similar manner but with different RNAi construct (EcR-TRiP). Females mated to prd-GAL4; EcR-TRiP knockdown males (Panel A, Knockdown) produced significantly fewer progeny (Panel A, ***p = 0.0007; N = 15–20) when compared to their genetically matched sibling controls (Panel A, Control). To determine the extent of knockdown of EcR when EcR-TRiP is driven through prd-GAL4, proteins from male accessory glands of control (Sb; UAS-EcR-TRiP) and knockdown (prd-GAL4; UAS-EcR-TRiP) males were isolated and western blotting was performed with anti-EcR, anti-USP antibodies (Panel B). Protein samples from knockdown males had detectable levels of EcR but yet significantly reduced when compared to control (EcR panel). However, USP levels were comparable between control and knockdown samples (USP panel). Blots were probed with α-tubulin as a control for protein loading in control and knockdown. (PDF) [file pgen.1006788.s003.pdf]

# Accessory Gland

USP control

USP-

miRNA

50%

75%

100%

USP

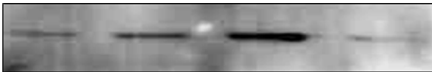

$\beta$ -actin

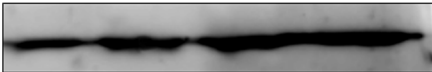

Supplement: S4 Fig — In western blots, the extent of USP knockdown was quantified by running serial dilutions to the level of 50% (lane 50%),75% (lane 75%) and 100% (lane 100%), of accessory gland proteins from ten USP control males in parallel with accessory gland extracts equivalent to ten USP knockdown males (lane USP-miRNA). Three independent sets of samples were analyzed to confirm the level of knockdown. Blots probed with β-actin suggest the extent of protein loading in control and knockdown. (PDF) [file pgen.1006788.s004.pdf]

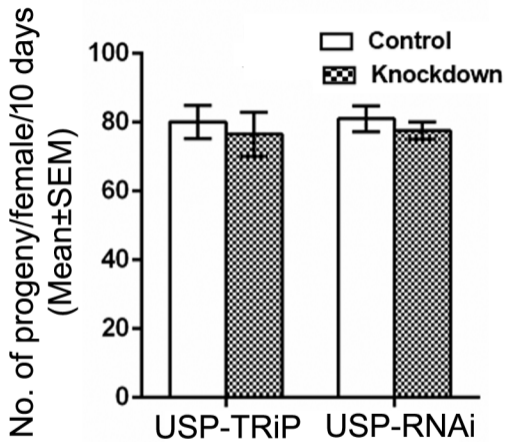

Supplement: S5 Fig — To confirm that the depletion of USP has no impact of male fertility, USP was knocked down in a similar manner but with different RNAi constructs (USP-TRiP and USP-RNAi). The number of progeny produced over a period of 10 days by females to prd-GAL4; USP-TRiP knockdown males (p = 0.7051; N = 15–20) and those mated to prd-GAL4; USP-RNAi knockdown males (p = 0.4711; N = 15–20) was comparable to their genetically matched controls (Control). (PDF) [file pgen.1006788.s005.pdf]

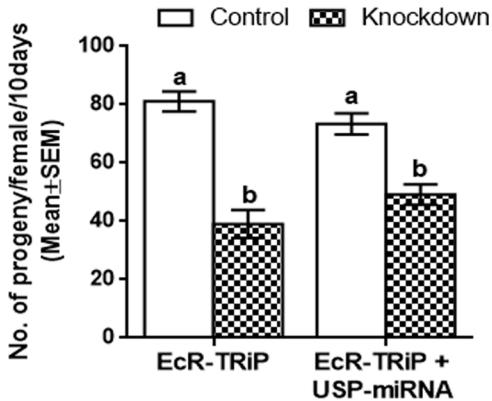

Supplement: S6 Fig — We hypothesized that if USP were to be involved in male fertility, depletion of USP in EcR-TRiP background should further enhance the fertility phenotype of EcR-TRiP. To test if this is the case, we generated a double knockdown of USP as well as EcR by driving the expression of both USP-miRNA and EcR-TRiP (EcR-TRiP+USP-miRNA) through prd-GAL4 and analyzed the fertility of females mated to these males in comparison to those mated to EcR-TRiP knockdown males. In both groups, females mated to knockdown males (Knockdown) produced fewer progeny when compared to those mated to their genetically matched sibling controls (***p<0.0007). However, the number of progeny produced over a period of 10 days by females mated to USP and EcR double knockdown males was comparable to that of EcR-TRiP knockdown mates (p = 0.2589). Values represented here are Mean±SEM involving at least 15–20 mated females per group and assays were repeated at least twice. The bars having same letter are statistically non-significant (control Vs control: p = 0.5493; single knockdown Vs double knockdown: p = 0.2589) and significantly differ from those with a different letter (Control Vs knockdown: ***p = 0.0007). (PDF) [file pgen.1006788.s006.pdf]

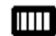 Control  
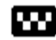 Knockdown

A. Fecundity

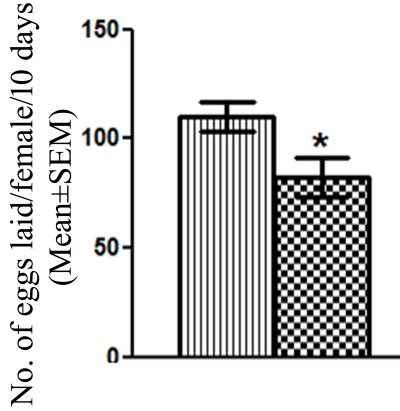

B. Fertility

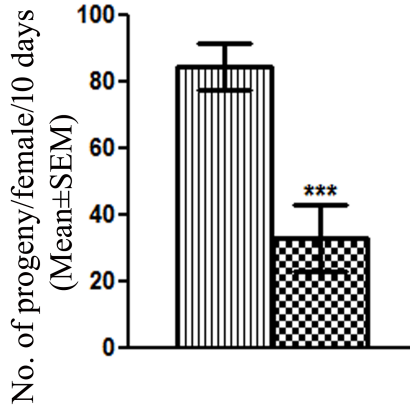

C. Hatchability

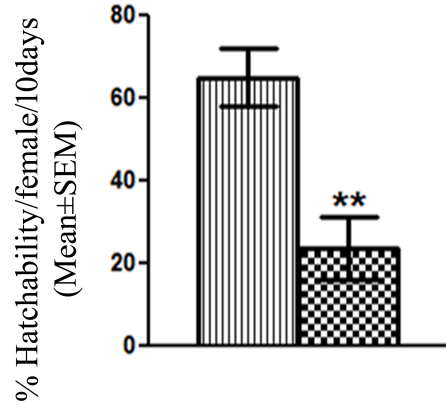

EcR-TRiP

Supplement: S7 Fig — To assess the reproductive performance, we mated 3-5days old virgin females with control or knockdown males and assessed the number of eggs laid (A, Fecundity), number of progeny produced (B, Fertility) by mated females and the proportion of eggs reaching adulthood (% hatchability) over a period of 10 days ASM. Females mated to EcR-TRiP knockdown males laid significantly fewer eggs (*p = 0.03; N = 10–15). Similarly, females mated to knockdown males produced significantly fewer progeny (***p = 0.0007; N = 10–15) when compared to control. This reduction in progeny production is a consequence of reduced egg laying as well as significantly reduced hatchability of these laid eggs (**p = 0.002; N = 10–15) when compared to those of controls. (PDF) [file pgen.1006788.s007.pdf]

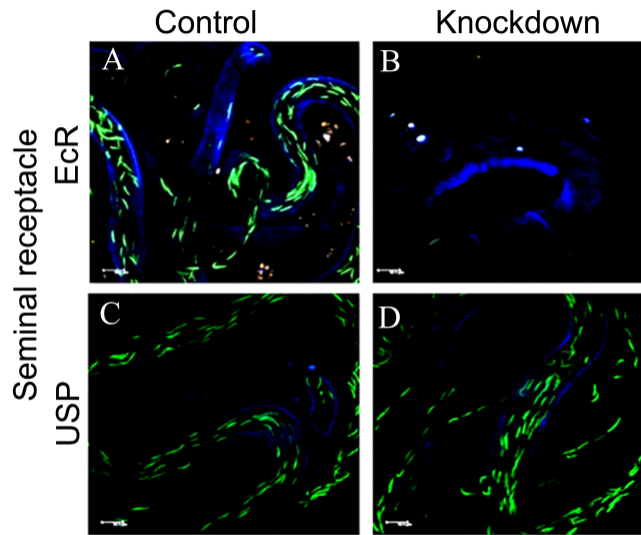

**E**

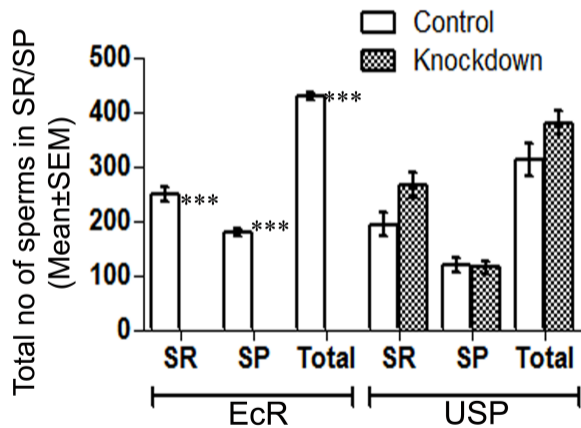

Supplement: S8 Fig — Panels A-D show sperm storage (green) at 2h ASM in seminal receptacle of females mated to EcR control (A), knockdown (B), USP control (C) or knockdown (D). Panel E shows the number of sperm stored in seminal receptacle (SR), Spermathecae (SP) and the total sperm in storage of females mated to EcR or USP knockdown males in comparison to sperm storage levels in controls. EcR control, USP control and knockdown had comparable number of sperm in storage while EcR knockdown mates were devoid of sperm in their sperm storage organs (***p< 0.0001, N = 5). (PDF) [file pgen.1006788.s008.pdf]

Control

Knockdown

EcR

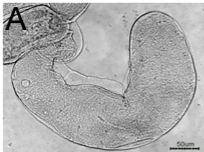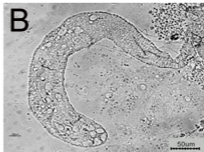

USP

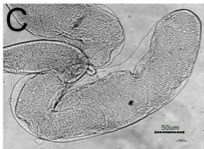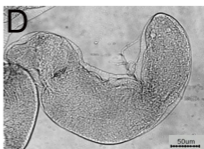

Supplement: S9 Fig — Accessory glands from 3–5 days old control and knockdown (EcR or USP) males were dissected out in normal saline (10 replicates for each group) and observed under a phase contrast microscope. In EcR control (Panel A), accessory gland appeared bulged and filled with secretions. In the EcR knockdown (Panel B), accessory gland was reduced and flaccid. Panels C and D represent accessory glands from USP control and knockdown, respectively, and both are filled with secretions as in EcR controls. Images were taken at 200X under an inverted microscope. (PDF) [file pgen.1006788.s009.pdf]

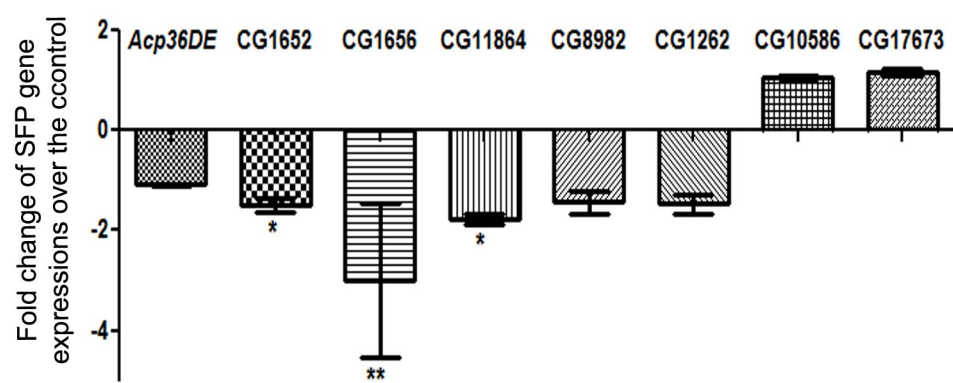

Supplement: S10 Fig — Analysis of transcript levels through qPCR revealed significant down-regulation (*p<0.05) of secondary cell derived Acps (CG1652, CG1656) and main cell derived Acp (CG11864) in accessory glands from EcR-TRiP knockdown males The transcript levels of Acp36DE, Ovulin (CG8982), Acp62F (CG1262), Seminase (CG10586) and SP (CG17673) did not differ from controls. The ΔCt values were determined through normalization against RPL32, which was used as an internal control for the quality of the template. Experiment was carried out thrice for each transcript. (*p = 0.02, **p = 0.007). (PDF) [file pgen.1006788.s010.pdf]

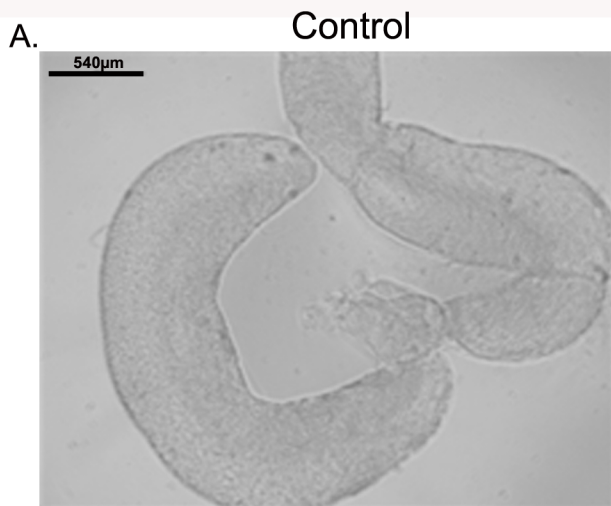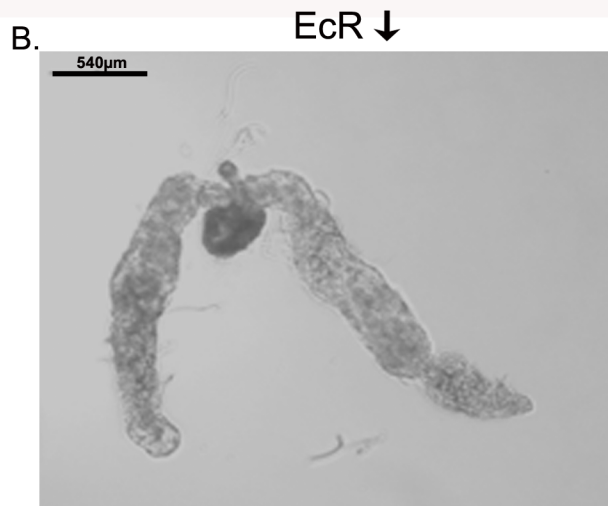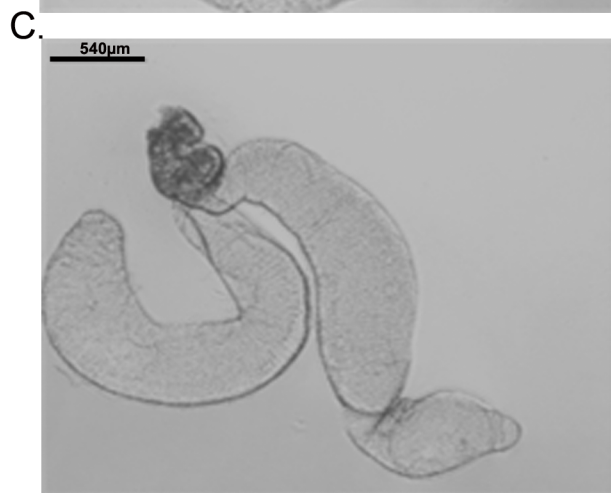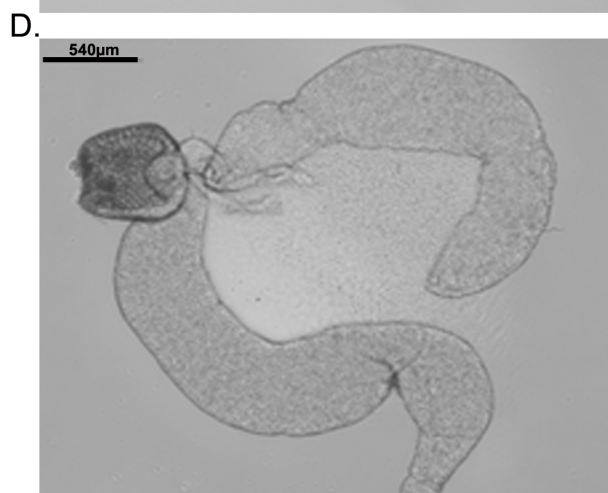

Supplement: S11 Fig — Accessory glands from 3–5 days old control and knockdown (EcR or USP) males were dissected out in normal saline (10 replicates for each group) and observed under a phase contrast microscope. In the EcR control (Panel A), the accessory gland appeared bloated and filled with secretions. In the EcR knockdown (Panel B), the accessory gland was highly reduced and the lumen appeared empty. Accessory glands from EcR knockdown males over expressing P35 (Panel C) or Diap1 (Panel D) were filled with secretions as in EcR controls. Images were taken at 100X under an inverted microscope. (PDF) [file pgen.1006788.s011.pdf]

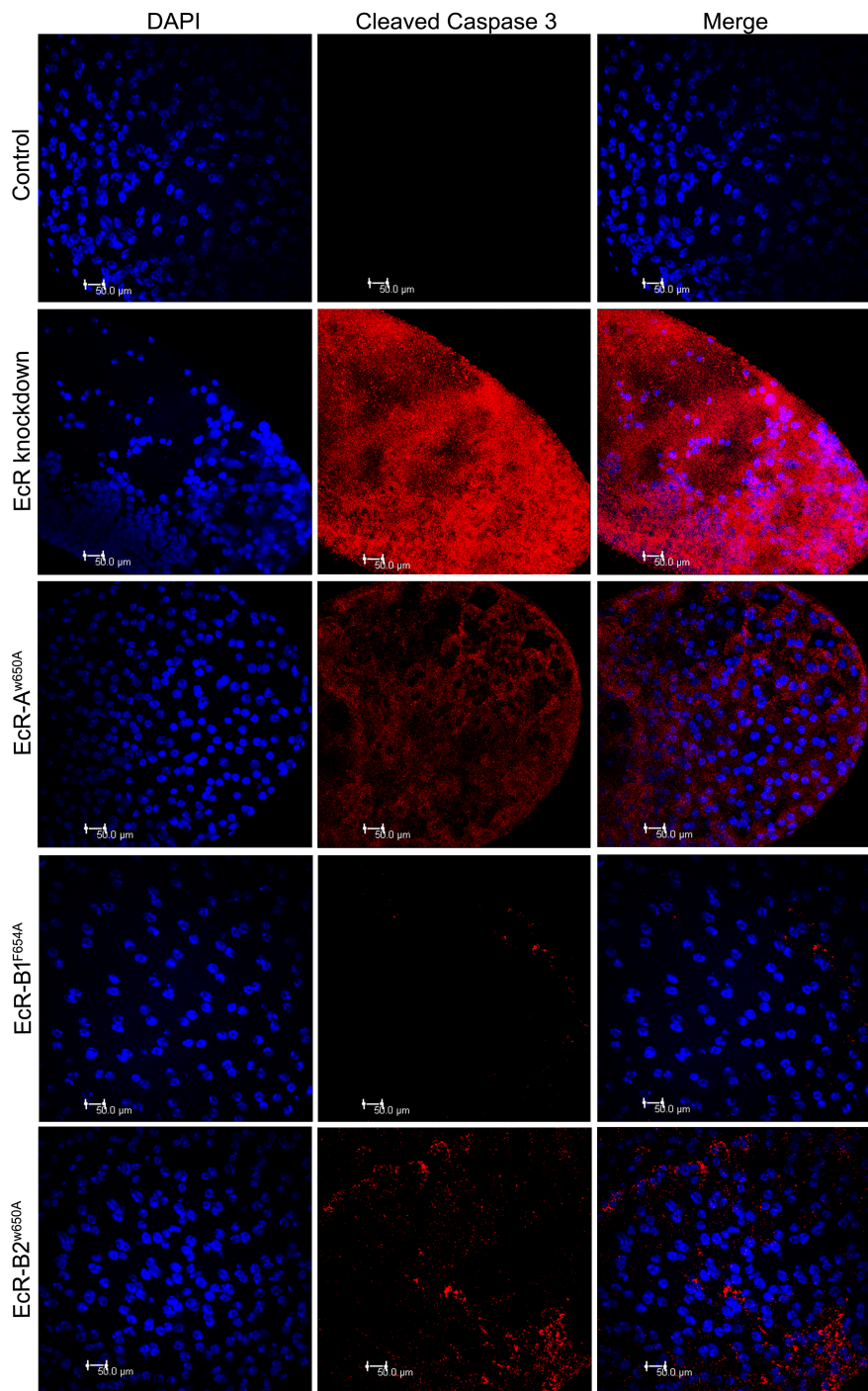

Supplement: S12 Fig — To examine if inhibition of any of the EcR isoforms leads to induction of apoptosis in accessory glands, tissues from EcR-AW650A, EcR-B1F645A, EcRB-2W650A were immunostained with antibodies for cleaved Caspase 3 and were compared with the tissues from the control (Control) and EcR-miRNA (EcR knockdown) immunostained in parallel. The DAPI panels show the nuclei (Blue in color; DAPI) and cleaved Caspase 3 panels represent the immunoreactivity in Red color (cleaved Caspase 3) while the overlay shows the nuclei and cleaved Caspase 3 immunoreactivity (Overlay). (PDF) [file pgen.1006788.s012.pdf]

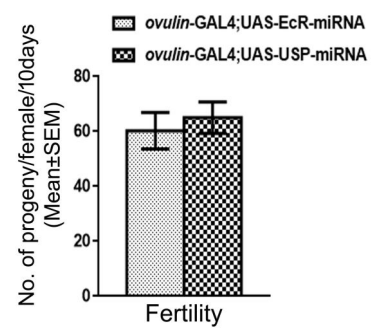

Supplement: S13 Fig — To test if EcR activity is required at an early or late stages of accessory gland development, UAS-EcR-miRNA or UAS-USP-miRNA were driven under the control of ovulin (An Acp gene) promoter based GAL4, which expresses specifically in the accessory glands at the late pupal stage after the formation of accessory glands. We did not detect significant reduction due to ovulin promoter driven EcR knockdown and the fertility of EcR knockdown mates were comparable to that of USP knockdown mates (p>0.1). (PDF) [file pgen.1006788.s013.pdf]
