# Supplementary material for: Functional male accessory glands and fertility in Drosophila require novel ecdysone receptor
Source: PLoS Genet. 2017 May 11;13(5):e1006788. doi: 10.1371/journal.pgen.1006788 (PMC5444863; doi:10.1371/journal.pgen.1006788)
Supplement: S2 Fig — To confirm the tissue specificity of prd-GAL4 expression, we crossed the prd-GAL4 to G-TRACE line carrying fluorescent protein reporters for both real-time expression of GAL4 (RFP) and transient expression of GAL4 during development (enhanced GFP (EGFP)). We analyzed the brain ganglia, genital disc as well as gonads from male larvae and testes as well as accessory glands (tissues in blue, stained with DAPI) from adult males generated from the above cross for RFP or EGFP signals under a confocal microscope. We detected RFP and EGFP signals only in adult male accessory glands but not in larval brain ganglia, genital disc, gonads and adult testes, indicating the accessory gland specificity of prd-GAL4 driver. (PDF) [file pgen.1006788.s002.pdf]

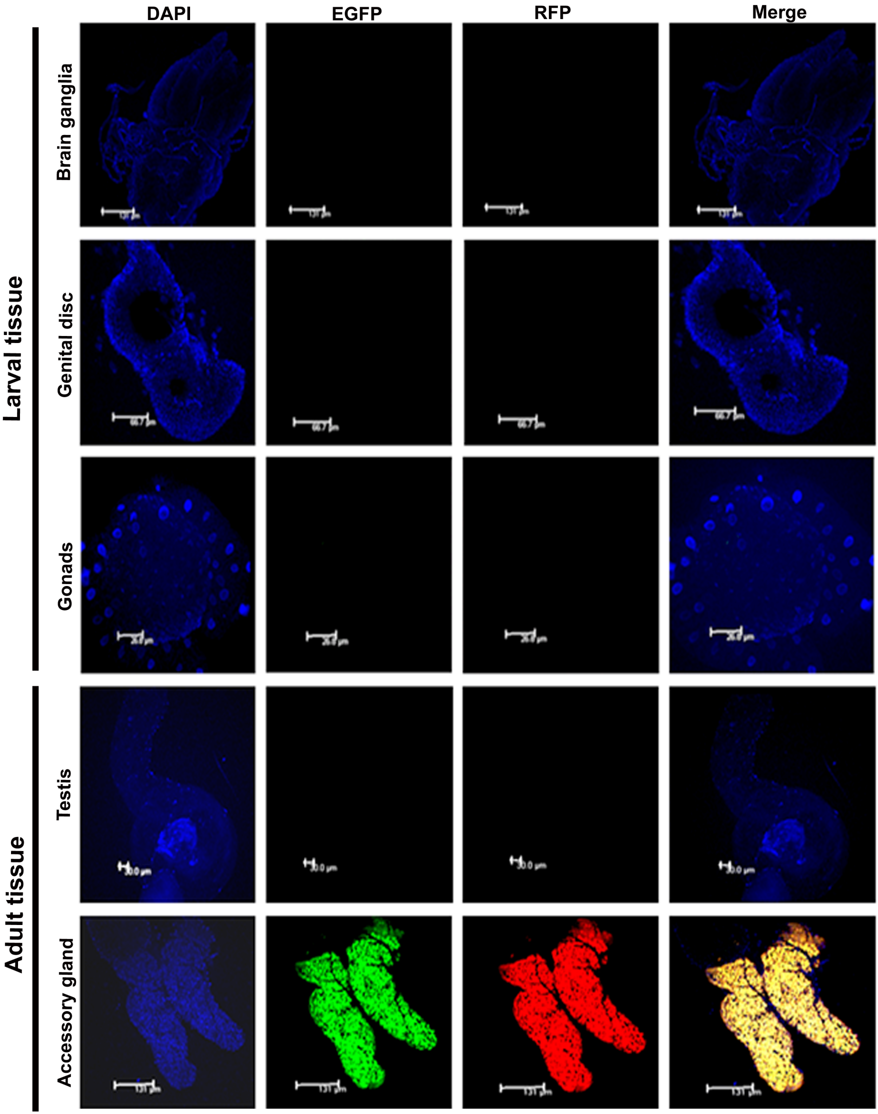
DAPI
EGFP
RFP
Merge
Larval tissue
Brain ganglia
Genital disc
Gonads
Adult tissue
Testis
Accessory gland
